# Efferocytosis drives myeloid NLRP3 dependent inflammasome signaling secretion of IL-1β to promote tumor growth

**DOI:** 10.3389/fimmu.2022.993771

**Published:** 2022-11-09

**Authors:** Cara Lang, Sohini Roy, Yu Wang, Diana Graves, Yaomin Xu, C. Henrique Serezani, Michael Korrer, Young J. Kim

**Affiliations:** ^1^ Department of Pathology, Microbiology & Immunology, Vanderbilt University, Nashville, TN, United States; ^2^ Department of Otolaryngology-Head and Neck Surgery, Vanderbilt University Medical Center, Nashville, TN, United States; ^3^ Department of Biostatistics, Vanderbilt University Medical Center, Nashville, TN, United States; ^4^ Center for Quantitative Sciences, Vanderbilt University Medical Center, Nashville, TN, United States; ^5^ Vanderbilt Center for Immunobiology, Vanderbilt University Medical Center, Nashville, TN, United States; ^6^ Department of Medicine, Division of Infectious Diseases, Vanderbilt University Medical Center, Nashville, TN, United States; ^7^ Vanderbilt-Ingram Cancer Center, Vanderbilt University Medical Center, Nashville, TN, United States

**Keywords:** inflammasome, efferocytosis, NLRP3, IL-1β, immunotherapy, tumor associated macrophage

## Abstract

Caspase-1 signaling in myeloid suppressor cells can promote T-cell independent cancer progression, but the regulation of inflammasome signaling within the highly heterogeneous myeloid population in the tumor milieu remains elusive. To resolve this complexity, single cell transcriptomic profile of Head and Neck Squamous Cell Carcinoma (HNSCC) identified distinct inflammasome-associated genes within specific clusters of tumor-infiltrating myeloid cells. Among these myeloid cells, the sensor protein, NLRP3, and downstream effector IL-1β transcripts were enriched in discreet monocytic and macrophage subtypes in the TME. We showed that deletion of NLRP3, but not AIM2, phenocopied caspase-1/IL-1β dependent tumor progression *in vivo*. Paradoxically, we found myeloid-intrinsic caspase-1 signaling increased myeloid survival contrary to what would be predicted from the canonical pyroptotic function of caspase-1. This myeloid NLRP3/IL-1β signaling axis promotion of tumor growth was found to be gasdermin D independent. Mechanistically, we found that phagocyte-mediated efferocytosis of dying tumor cells in the TME directly activated NLRP3-dependent inflammasome signaling to drive IL-1β secretion. Subsequently we showed that NLRP3-mediated IL-1β production drives tumor growth *in vivo*. Dynamic RNA velocity analysis showed a robust directional flow from efferocytosis gene-set ^high^ macrophages to an inflammasome gene-set ^high^ macrophage population. We provide a novel efferocytosis-dependent inflammasome signaling pathway which mediates homeostatic tumor cell apoptosis that characterizes chronic inflammation-induced malignancy.

## Graphical Abstract

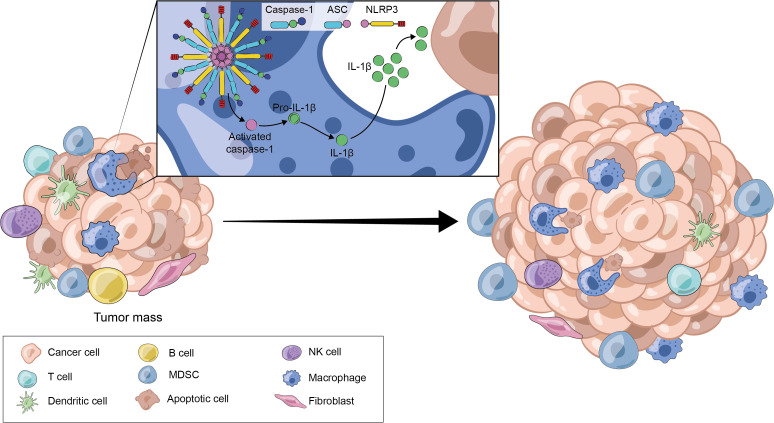

## Highlights

Dynamic single-cell profiling of human squamous cell carcinoma delineates specific efferocytosis gene set enriched tumor-infiltrating mononuclear phagocytes that transition to distinct myeloid cells with high upregulation of NLRP3.

Efferocytosis of tumor apoptotic debris activates NLRP3-dependent inflammasome signaling and IL-1β production, which drives tumor progression *in vivo.*

NLRP3/Caspase-1/IL-1β signaling axis within the tumor-infiltrating mononuclear phagocytes promotes tumor growth in GSDMD independent manner *in vivo*.

Myeloid intrinsic caspase-1 activity maintains myeloid cell survival in tumor microenvironment without induction of immune cell trafficking to the tumor site.

## Introduction

Chronic inflammation-associated immunosuppressive myeloid cells in the tumor microenvironment (TME) have been linked to poor clinical outcomes and failure of immunotherapy (1, 2), and the inflammasome complex has been proposed to play a central role in this process. The role of the inflammasome pathway in cancer has been contradictory and context-dependent (3). Our group and others demonstrated that myeloid intrinsic inflammasome activation is an important mechanism to promote tumor growth *in vivo* (4). The inflammasome pathway can be activated from invading pathogens or damage associated molecules (DAMP) through various sensor proteins (such as NLRP3, AIM2, etc.) to oligomerize the adaptor protein Apoptosis-associated speck-like protein containing a CARD (ASC) and to activate caspase-1, IL-1β, and IL-18 (5). Activated caspase-1 also cleaves gasdermin D (GSDMD) and its N terminal cleavage product inserts into the cell membrane to induce pyroptosis, an immunogenic cell death pathway (6). The Canakinumab Anti-inflammatory Thrombosis Outcomes Study (CANTOS) trial using IL-1β blocking canakinumab in cancer-free patients with atherosclerosis found that patients treated with canakinumab had overall lower cancer incidences and lower cancer-associated morbidity and mortality, and thus provides compelling evidence for a pro-tumorigenic role of chronic IL-1β driven inflammation in humans (7, 8).

However, an unanswered question centers around the regulatory mechanism of myeloid-intrinsic inflammasome activation driving this pro-tumorigenic phenotype. An under-characterized dynamic process in the TME is efferocytosis (9–12), a well-orchestrated and immunologically quiescent pathway of phagocytic clearance of apoptotic cells (AC) (13–15). In adults, homeostatic efferocytic clearance of apoptotic cells (10^6^ cells/day) (16, 17) induces the expression of an immunosuppressive and wound healing gene signature (high *IL10, IL4, TGFβ* and low *IL12)* in macrophages to regulate tolerance and aberrant activation of autoimmune responses (18–21). Mitogenic activity of tumor cells is characterized by apoptotic indices (apoptotic nuclei per 100 intact neoplastic cells) as high as 5-10% and may often correlate with poor prognosis (22–32). Early reports have demonstrated that tumor associated macrophages (TAM) recruited to the TME efficiently clear the tumor AC through efferocytosis, which may polarize myeloid cells towards tumorigenic and metastasis promoting M2 macrophages (9, 10, 33). While previous studies have reported efferocytosis in non-tumor milieu is associated with inflammasome activation (34, 35), the mechanistic relationship between efferocytosis of apoptotic tumor cells and inflammasome activation is currently not clear.

Here, we examined the tumor intrinsic factor that regulates myeloid inflammasome signaling in both human and murine tumors, and we provide evidence that mononuclear phagocytic-mediated efferocytosis of apoptotic cancer cells triggers NLRP3 dependent inflammasome activation and IL-1β secretion to promote tumorigenesis.

## Method details

### Induction of apoptosis and annexin V/PI staining

Apoptosis was induced in Cal27 cells by treating cells with 2uM Staurosporine for 15hrs. To induce apoptosis in MOC2 cells, they were incubated with 75uM AZD5582 for 20hrs.

Cells were harvested and washed in PBS and resuspended in 100ul 1X Annexin V staining buffer. Cells were stained with 5ul Annexin V (APC) for 15mins in the dark at RT. Cells were washed once in Annexin V buffer, stained with PI and analyzed by flow cytometry. This process regularly yielded 70-80% early apoptotic cells (Annexin V+PI-).

Efferocytosis experiments were strictly conducted with cells that were early apoptotic.

### Efferocytosis assay

For efferocytosis assay, depending on the experiments, macrophages were plated in either cell culture petridishes or two-well chambers (for confocal imaging). Either Annexin V+/PI- Cal27 or MOC2 cells were added to the macrophages for the indicated time points. Where mentioned, apoptotic cells were stained with PKH26 cell membrane labeling kit following manufacturer’s instructions. For RNA sequencing experiments, efferocytosis was stopped after 1hr (pulse) using ice cold PBS. Macrophages were vigorously washed with PBS to remove non-phagocytosed cancer cells. Fresh media was added for another 6hrs (chase) following which cells were harvested, stained with antibodies against CD11b and F4/80. Macrophages that had efferocytosed apoptotic cancer cells were isolated by flow sorting as CD11b+F4/80+PKH26+ cells and processed for bulk RNA sequencing. Where mentioned, macrophages were treated with small molecule inhibitors to target inflammasome pathway or block the uptake of apoptotic tumor cells. For confocal assays, macrophages were washed with 1X PBS and stained with WGA cell labeling dyes and Hoechst.

### Efferocytosis uptake assay

BMDM or human macrophages were incubated with Annexin V+/PI- MOC2 or Cal27 cells stained with PKH26+ dye following manufacturer’s instructions. After 1hr, macrophages were washed with ice cold DPBS to remove excess apoptotic cells that were not engulfed. Following this, macrophages were harvested and processed for analysis by either flow or confocal microscopy.

### Detection of inflammasome speck formation by confocal microscopy

Macrophages were grown in two-well chambers as described previously. Where mentioned, cells were treated with 1ug/ml LPS and/or 5ug/ml Nigericin as inflammasome agonists. Following efferocytosis, macrophages were washed with 1X PBS and stained with WGA594 for 5mins in 4°C. Cells were washed with 1x PBS and nuclei were stained using Hoechst dye in PBS for 5mins and analyzed by Zeiss LSM 800 with Airyscan at the VU CISR imaging facility. Data were analyzed and processed with the Zeiss Zen 2010 software. All confocal data were quantified using ImageJ software, and graphical illustrations were made using GraphPad Prism software as mentioned later.

*Detection of inflammasome “speck” formation in myeloid cells from murine tumors*

MOC2 cells (200,000) were subcutaneously injected into the flank of ASC Citrine/LysmCre mice. Where mentioned, mice were treated with MCC950 I.P. (15mg/kg body weight) daily for first three days and then every alternate day for rest of the study. Tumors were harvested after 15days and digested to get single cells. F4/80+ cells were isolated using magnetic bead separation and immediately processed for analysis by confocal microscopy. Hoechst was used to stain the nucleus.

### *In vivo* tumor model

**Figure 1:**
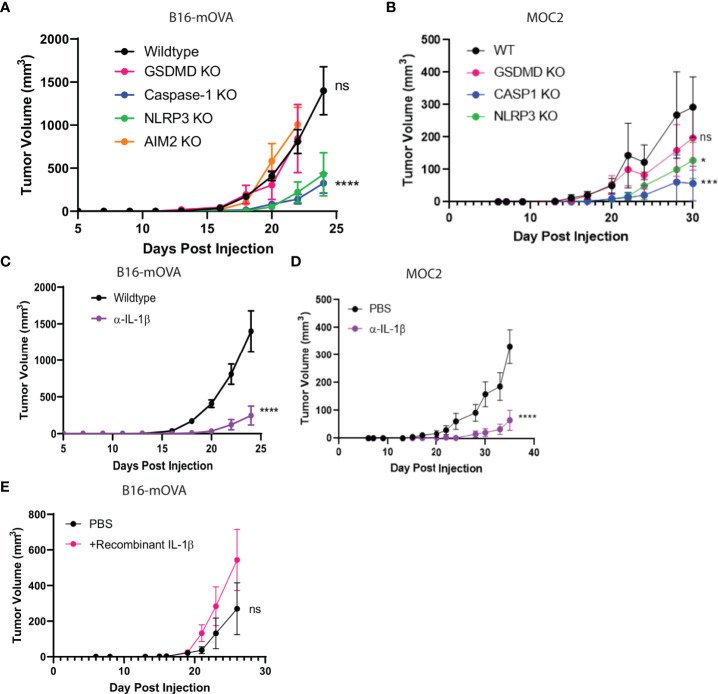
NLRP3/Casp-1/IL-1β axis drives tumor growth. **(A, B)** The inflammasome sensor, NLRP3, and caspase-1 drive tumor growth. B16-mOVA **(A)** and MOC2 **(B)** tumor growth shown in GSDMD, caspase-1, AIM2, and NLRP3 global knockout mice as compared to tumor volume in WT mice. (n=5 mice/group). **(C, D)** IL-1β promotes B16-mOVA **(C)** and MOC2 **(D)** tumor growth as shown by a reduction in tumor volume upon IL-1β depletion compared to control PBS treated mice. Mice treated with 100 µg of IL-1β depleting monoclonal antibody or control 100 µl PBS 2x/week (n= 10 mice/group). **(E)** Increased IL-1β drives B16-mOVA tumor growth. Mice treated with 5 ng recombinant IL-1β or control 100 µl PBS 2x/week (n=5 mice/group). All statistics represent two-way ANOVA and all error bars represent SEM. (*p < 0.05, ***p < 0.001, ****p < 0.0001; ns, not significant).

**Figure 2:**
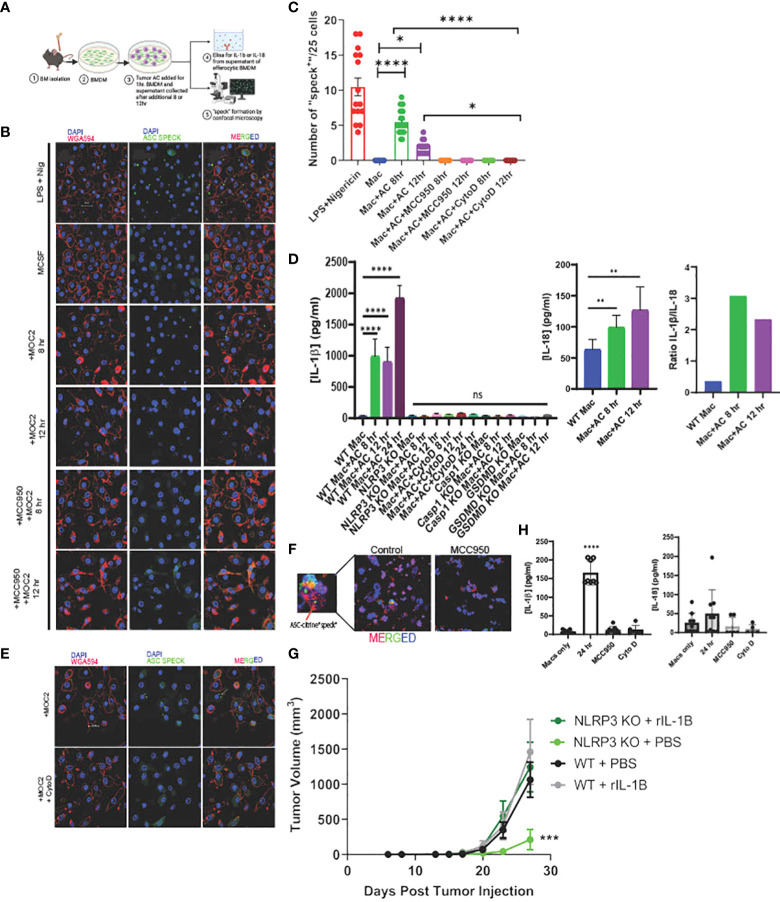
Efferocytosis induces inflammasome assembly and IL-1β production in macrophages in NLRP3-dependent manner. **(A)** Schema of efferocytosis induced inflammasome activity using ASC Citrine/LysMCre BMDM. **(B)** Efferocytosis of tumor AC induces inflammasome “speck” formation in BMDM in a time dependent manner. BMDM from ASC Citrine/LysMCre mice were incubated with AC for 1hr. Non-engulfed AC were then washed off and “speck” formation was assessed using confocal microscopy at mentioned time points. BMDM were treated with 1ug/ml LPS+ 5ug/ml Nigericin (top) for four hours as a positive control. Treatment with the NLRP3 small molecule inhibitor MCC950 at a concentration of 5uM overnight abolished the effect. The first panel shows macrophages (red). The second panel shows “specks” (green). The third panel shows merged images of macrophages with inflammasome “specks” formation. Scale bar, 20µm. **(C)** Graphical representation showing quantification of “speck” positive cells under conditions shown in **(B)**. **(D)** Graphical representation of ELISA assay showing secretion of IL-1β and IL-18 from BMDM post treatment with AC at time points=8, 12 and 24hr. Ratio of IL-1β and IL-18 post efferocytosis. IL-1β was not detected post efferocytosis in NLRP3 KO BMDM or when treated with AC engulfment inhibitor Cytochalasin D at a concentration of 10uM. **(E)** BMDM treated with Cytochalasin D abrogated “speck” formation following efferocytosis. The top row represents BMDM incubated with AC. The bottom row shows BMDM treated with 10uM Cytochalasin D for 1hr prior to AC uptake. Scale bar, 20µm. **(F)** F4/80^+^ TAM (red) isolated from MOC2 tumors from ASC Citrine/LysmCre mice show inflammasome activation (green) *in vivo*. Tumor bearing mice treated with MCC950 I.P. (15mg/kg body weight) daily for first three days and then every alternate day for rest of the study) failed to activate inflammasome “specks” *in vivo*. Inset shows magnified image of TAM with inflammasome “speck” formation. DAPI was used to stain nuclei. **(G)** Recombinant IL-1β treatment rescues B16-mOVA tumor growth in NLRP3 KO mice. Mice treated with 5 ng recombinant IL-1β 2x/week (n=5 mice/group). **(H)** PBMC were differentiated to macrophages using 100ng/ml M-CSF for 7days and then subjected to efferocytosis with tumor AC. IL-1β but not IL-18 is secreted from human macrophages post efferocytosis. MCC950 (5uM, overnight) and Cytochalasin D (10uM, 1hr before uptake) blocked IL-1β secretion in efferocytic macrophages. Data for “speck” formation and ELISAs shown from independent experiments with n=3-5 mice. Graphs shown as mean ± SEM. (*p < 0.05, **p < 0.01, ***p < 0.001, ****p < 0.0001).

To look at tumor growth in wildtype C57Bl/6 mice or knockout mice, 1x10^5^ B16 or Moc2 cells were injected subcutaneously into the flanks of the mice. Tumors were measured 4x/week with calipers and tumor volumes were estimated using the formula V (cm^3^) = 3.14 × [largest diameter × (perpendicular diameter)^2^]/6. Mice were sacrificed when the tumors measured 2 cm in the largest diameter. To generate chimeric mice, 5x10^6^ bone marrow cells from gasdermin D^-/-^ mice or wildtype C57Bl/6 mice were adoptively transferred to lethally irradiated (10 Gy) gasdermin D^-/-^ or wildtype C57Bl/6 mice. 8 weeks after bone marrow transfer, engraftment was confirmed by the presence of CD45+ PBMCs. After engraftment confirmation, B16 or MOC2 tumor cells were injected and monitored as mentioned above. Depleting anti-PD-1 (100 µg/mouse), anti-IL-1β (100 μg/mouse) was injected intraperitoneally twice a week in WT B16 and MOC2 tumor bearing mice to assess alterations in tumor growth rate (**Figures 1C, D**). or r Recombinant IL-1β (1 μg/mouse) was injected intraperitoneally twice a week for the NLRP3 KO tumor rescue experiments (**Figure 2G**) in some cohorts. For *in vivo* myeloid persistence experiments, CD45.1 ^+/-^ mice were lethally irradiated with 10 Gy and reconstituted with 5x10^6^ CD45.1^+/+^ wildtype bone marrow cells and 5x10^6^ CD45.2^+/+^ caspase-1 knockout bone marrow cells 12 hours post irradiation. 8 weeks post irradiation 1x10^5^ B16 tumor cells were injected subcutaneously. 4 weeks post tumor injection, tumors were harvested, and flow cytometry was performed to identify tumor infiltrating myeloid cell populations.

### ELISA

BMDM or human macrophages were cultured and efferocytosis performed as described earlier. 200 µl of supernatant from each well of cultured efferocytic mouse macrophages of different treatment conditions were collected at different time points for measurement of IL-1β and IL-18 using ELISA kits without dilutions. Supernatant from caspase 1 knockout and wildtype B16 cell lines was used to confirm caspase 1 knockout. Each assay had duplicated wells for the standard curve and triplicated wells for the samples. Concentration was calculated from the standard curve using the recombinant cytokines, per the manufacturer’s instructions.

### Flow cytometry

For analysis of tumor-infiltrating populations, mice were sacrificed at indicated time points and tumors were harvested, disrupted in small pieces, and incubated with collagenase and DNase for 1 hour at 37°C. Single-cell suspensions were prepared by mincing the tumors through a 70-μm cell strainer (BD Biosciences). Cells were first incubated with live/dead fixable yellow dead cell stain (Thermo Fisher Scientific, L34959) in PBS for 15 minutes at 4˚C. Subsequently, cells were incubated with monoclonal antibodies for 15 minutes at 4˚C. Samples were acquired on a BD FACSCelsta flow cytometer, and results were analyzed using the FlowJo software. For intracellular staining, the cells were surface stained and then fixed and permeabilized for 30 minutes with Fix/Perm buffer at 4˚C. Following fix/perm, cells were intracellularly stained for 30 minutes in perm buffer at 4˚C. For IFN-γ, perforin, and granzyme, cells were incubated with PMA/ionomycin (Cell stimulation cocktail, Invitrogen) and monensin (BioLegend) for 4 hours at 37°C prior to antibody staining.

### Suppressive myeloid cell generation

Wildtype C57Bl/6 or knockout bone marrow cells were obtained from the femur and tibia, cultured in RPMI-1640 supplemented with 10% FBS, 1% penicillin/streptomycin, 1% HEPES, 1% Glutamax, and 0.1% beta-mercaptoethanol. 10 ng/ml GM-CSF was added to induce suppressive myeloid cells. After 4 days, non-adherent cells were collected and CD11b+ cells were positively selected by magnetic-activated cell sorting.

### T cell proliferation assay

CD3+ T cells were isolated from the spleens of wildtype C57Bl/6 or knockout mice by magnetic activated cell sorting and stained with 5 µM CFSE for 5 minutes in PBS + 5% FBS. Cells were washed twice with PBS + 10% FBS prior to plating. Isolated CD3+ T cells were co-cultured with bone marrow derived MDSCs at indicated ratios on 96 well plates with anti-CD3/CD28 stimulation beads. The percentages of proliferating CD3+ T cells were determined by CFSE dilution and PD-1 expression was assessed by flow cytometry analysis.

### Viability assay

Wildtype and knockout MDSCs were generated as stated above. MDSCs were plated at a concentration of 100,000 cells/well in 100 μl in a 96 well plate. Certain groups were also treated with LPS or tumor conditioned media. Cells were treated with 5 um green caspase 3/7 dye and red annexin V dye. Fluorescing cells were counted using using Incucyte FLR live imager and software.

### Caspase-Glo assay

B16 tumors were grown in wildtype and caspase 1 knockout C57Bl/6 mice. Tumors and spleens were harvested on day 16 post injection. CD11b+ cells were positively selected using magnetic activated cell sorting. Cells were plated at a concentration of 100,000 cells/well in 100 ul in a 96 well plate. Caspase 1 activity was determined using the Caspase 1 glo kit (Promega) according to manufacturer protocol.

### Single cell RNA sequencing

Head and neck cancer patient samples were weighed and cut into pieces prior to digestion using human tumor digestion mix for 1hr on the tumor dissociator. Cells were then passed through a 70uM cell strainer to remove debris and centrifuged at 300g for 5mins. RBC was lysed with RBC lysis buffer. Cells were then centrifuged again and resuspended at 600cells/uL PBS and dropped off for Chromium Single Cell 3’ Library construction (10X Genomics) and sequencing at VANTAGE sequencing facility at VU following the manufacturer’s instructions. Libraries were sequenced on an Illumina HiSeq4000 and mapped to the human genome (build GRCh38) by CellRanger (Version 3.0) (36).

### Bulk RNA sequencing

CD11b+ myeloid cells were flow sorted from previously untreated HNSCC tumors and matched blood (n=7). Briefly, tumors were digested using human tumor digestion buffer following manufacturer’s instructions to obtain single cells. Following this, cells were stained with live dead dye, CD45, CD11b, CD3 and NK1.1. RNA was isolated from sorted CD11b+ cells using Trizol and used for sequencing at VANTAGE next generation sequencing facility at VU.

Mouse BMDM were incubated with Annexin V+/PI- Cal27 cells stained with PKH26+ dye for 1hr. After this, macrophages were washed with ice cold DPBS to remove apoptotic tumor cells that were not engulfed by the BMDM. After additional 6hr, macrophages were harvested and processed for flow sorting to obtain BMDM that had engulfed AC (PKH26+). RNA was isolated using Trizol and used for sequencing.

### Single-cell RNA-Seq data processing

The raw FASTQ files were demultiplexed and aligned with STAR (37) algorithm imbedded in CellRanger package. The raw unique molecular identifier (UMI) count matrix with the cell barcodes and the feature list for each sample was extracted and imported into R environment with Seurat 4.0 (38) for downstream analysis. For quality control, cells with UMI less than 1500 or greater than 15000 or mitochondrial-derived UMI counts over than 10% were excluded. Potential doublets were further checked and removed with Scrublet (39). To factor out the technical factors including sequencing depth, normalization and variance stabilization of UMI matrix was performed with SCTransform function, by considering the total UMI counts and percentage of mitochondrial-derived UMIs as confounders, fitting in Gamma-Poisson Generalized Linear Model, with considering the top 5000 variable features after ranking by residual variance. Data from each sample was then integrated in Seurat as described in Stuart etal, (40). During this process, we first got top 5000 variable genes as potential anchors with FindIntegrationAnchors function of Seurat. And then use IntegrateData function to integrate data. The technical batch effect between samples was regressed out during this step. Principal component analysis (PCA) was performed on the 5000 genes integrated matrix to reduce the dimensionality of the scRNA-Seq dataset, and top 50 PCs were chosen to keep more features of the dataset. The main cell clusters were identified with the FindClusters function in Seurat. Two-dimensional representation of the clusters were shown with tSNE or UMAP plots.

### Major cell type annotation

We use reference dataset-based annotation strategy to annotate the major cell types at single cell resolution. A fine annotated single cell RNAseq dataset (41) was downloaded at the NCBI GEO depository under the accession number GEO: GSE127465. Raw matrices of all human samples were imported into R environment and prepared as Seurat object by following the standard procedure (https://satijalab.org/seurat/articles/get_started.html). Meta data with the annotated cell types information were used as guideline of the reference. FindTransferAnchors and TransferData functions were performed sequentially to annotate our dataset with the reference.

Since the reference dataset was generated from human non-small-cell lung cancer samples and used different single cell generating platform (InDrop), to validate the robustness of the annotation approach, canonical cell markers of different major cell types were manually checked. The cell types with small cell numbers (RBCs, n = 5) were excluded from the downstream analysis.

### Myeloid cell subtypes annotation

To further annotate the myeloid population, subset of myeloid cells, including MoMacDC supercluster, pDCs, and Mast cells were extracted from the total dataset (due to small number, n = 12, Neutrophils were not included for further investigation). PCA and clusters were reanalyzed on the subsets of myeloid cells.

Pan-cancer level single myeloid cells were investigated across multiple cancer types and showed systematic view of the composition of tumor-infiltrating myeloid cells (42). We downloaded the expression data of the pan-cancer myeloid cells from Gene Expression Omnibus (GEO: GSE154763) as the reference dataset. Following the same workflow as described above, we prepare the integrated dataset as Seurat object and add in the meta data, including the myeloid cell annotation information.

Instead of the label transferring methods in Seurat, we used a multiple correspondence analysis (MCA) based technique, called Cell-ID (43) to annotate the myeloid cells in our dataset. Cell-ID can project the cells and genes in same low-dimensional space and therefore, can annotate single cell transcriptomes and report the gene signatures meanwhile. Cell-ID was approved to be robust even when there were batch effects, different donors, model organisms, tissues of-origin and single-cell omics protocols, which is ideal to be used as the tool for our myeloid cell annotation with pan-cancer myeloid cell reference data.

MCA dimensionality reduction were performed for both reference dataset and our myeloid cells. First, gene signatures of each myeloid cell types from the reference pan-cancer myeloid cells were extracted with GetGroupGeneSet function from Cell-ID using the first 50 dimensions. Then group-to-cell matching and label transferring across datasets strategy were adopted for annotation with RunCellHGT function in Cell-ID. If no significant hits are found, a cell will label as “Other”. Marker genes related to each sub-types of myeloid cells were manually reviewed and compared with the reference datasets. Similarity matrix defined based on the proportion of the shared marker genes between reference and our dataset in total number of marker genes showed each sub-types in our dataset have highest similarity with the same subtypes in reference dataset (data not shown). Per-cell functional enrichment analysis were performed by checking custom defined gene signatures (e.g. inflammasome and efferocytosis pathways) or genesets from MsigDB (44, 45).

### Marker gene identification

The cell type specific marker genes were identified by identifying preferentially expressed genes in certain cell type or differentially expressed genes between tumor- and blood-derived cells using the FindAllMarkers or FindMarkers function in Seurat with “MAST” method (46) by setting min.pct = 0.25, logfc.threshold = 0.25.

*Bulk RNA-Seq data analysis*

Raw fastq files were mapped to reference genomes (GRCh38 or GRCm38) with STAR software. Gene reads count were calculated with FeatureCounts (47) in R. Gene by sample matrix were integrated and performed the differential gene expression analysis workflow as described at https://bioconductor.org/packages/release/bioc/vignettes/DESeq2/inst/doc/DESeq2.html, mainly using DESeq2 package (48).

### Pathway analysis

GO and KEGG enrichment analysis was performed on DE genes with clusterProfiler (49). Pre-ranked GSEA was performed based on the ranking of gene list from the DE analysis using fgsea package (50). Single-sample-GSEA was conducted with the GSVA package (51). Differences of the ssGSEA enrichment scores of the gene sets between different sample or cell groups or clusters were calculated with a linear model offered by the Limma package (52). Functional enrichment analysis was also performed through the use of IPA (QIAGEN Inc., https://www.qiagenbioinformatics.com/products/ingenuitypathway-analysis).

### RNA velocity analysis

Spliced, unspliced and ambiguous matrices were extracted from the bam file generated by CellRanger for each sample and saved into loom file by velocyto (53). Guided with the expression matrix in seurat object as described above, these three matrices were integrated, and filtered for the low quality cells. RNA velocity analysis was carried out for the myeloid cells with scVelo (54) using the dynamical model for more consistent velocity estimates and better identification of transcriptional states compared with the stochastic model. The embedding information was inherited from the expression analysis to have the consistent dimension reduction plot. To portray the cell transitions between subclusters of myeloid cells, Partition-based graph abstraction (PAGA) was used for trajectory inference based on velocity-inferred directionality (55). Cluster-specific top-likelihood genes in the dynamic model were identified for each cluster to find the potential drivers that showed evident dynamic behavior.

### Statistical analyses

All the graphical illustrations and statistical tests were performed using Prism-6 software (GraphPad software, Inc., La Jolla, CA). All data reported in graphs are expressed as mean ± standard error of mean (SEM) unless otherwise mentioned and were compared using unpaired student *T*-test or ANOVA where mentioned. p values were considered statistically significant when less than 0.05. All experiments were repeated at least 3 times unless specified. *P <.05; **P <.005; ***P <.0005. ns=not significant.

## Results

### Single-cell transcriptomic analysis shows enrichment of inflammasome pathway genes among 13 distinct clusters of human tumor-infiltrating myeloid cells

**Figure 3:**
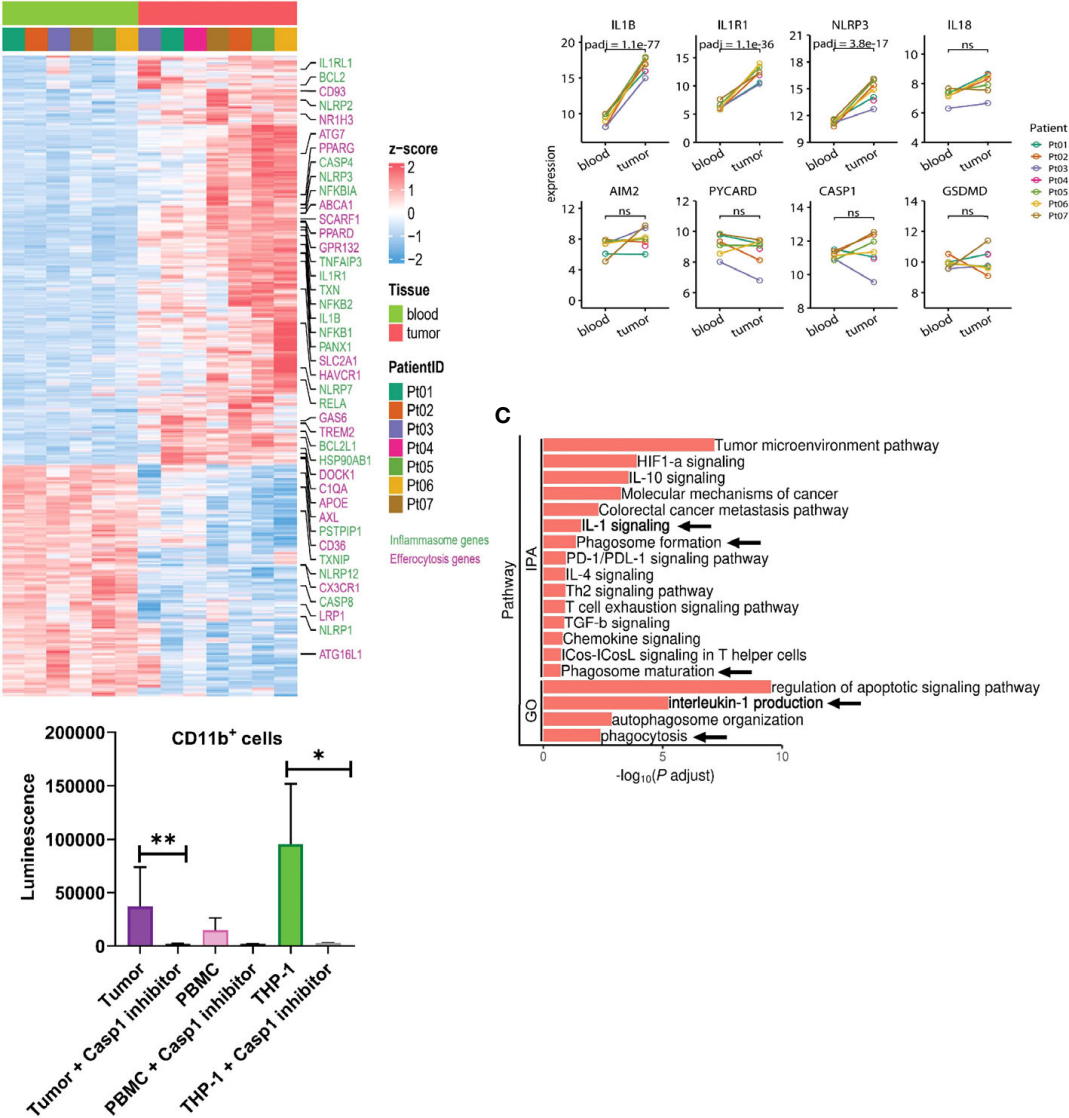
IL-1β signaling pathways are enriched in tumor infiltrating myeloid cells in human HNSCC. **(A)** Heatmap showing differential gene expression profile between CD11b^+^ myeloid cells flow sorted from matched blood or tumor from 7 HNSCC patients as measured by bulk RNA-seq. **(B)** Differential inflammasome signature in tumor and blood myeloid cells (n=7). **(C)** Ingenuity pathway (IPA) and gene ontology (GO) analyses showing enrichment of IL-1 signaling in tumor infiltrating myeloid cells over those from blood. **(D)** Caspase 1 activity is upregulated in tumor infiltrating CD11b^+^ myeloid cells compared to circulating blood CD11b^+^ cells (n=3). Statistics represent paired t-test and error bars represent SD (D). (*p < 0.05, **p < 0.01; ns, not significant).

To better characterize regulatory aspects of myeloid inflammasome signaling within the tumor, we initially performed bulk RNA sequencing of CD11b^+^ myeloid cells sorted from human head & neck squamous cell carcinoma (HNSCC) tumors with matched peripheral blood mononuclear cells (PBMC) (**Figure 3A**, **Supplementary Figures S1A–C**). We noted differentially increased inflammasome complex transcripts, including *NLRP3* and *IL1B* genes in the tumor-infiltrating myeloid cells compared to those in circulation (**Figures 3A, B**, **Supplementary Tables S1**, **S2**). Ingenuity Pathway Analysis (IPA) and Gene Ontology (GO) algorithms identified IL-1 signaling and IL-1 production pathways among the most significantly altered in tumor-infiltrating myeloid cells (**Figure 3C**). Furthermore, not all the transcripts associated with the inflammasome complex were enriched in the tumor, such as *IL18*, *AIM2, PYCARD*, and *GSDMD* (**Figures 3A, B**). The increased *NLRP3* and *IL1B* transcripts correlated with increased biochemical caspase-1 activity in the tumor infiltrating myeloid cells compared to those isolated from PBMC from cancer patients (**Figure 3D**). Our IPA and GO analyses further showed that in the tumor infiltrating CD11b^+^ cells, several tumor promoting and immune suppressive pathways are enriched and mobilized, including ‘tumor microenvironment pathway’, IL-10/IL-4/TGFb signaling, T cell exhaustion pathway (**Figure 3C**). Interestingly, pathways pertaining to phagocytosis, phagosome maturation and autophagosome organization were also upregulated in the tumor derived myeloid cells compared to their peripheral matched counterpart. This is also in agreement with existing reports that autophagic pathway is important for IL-1β secretion (56–59).

**Figure 4:**
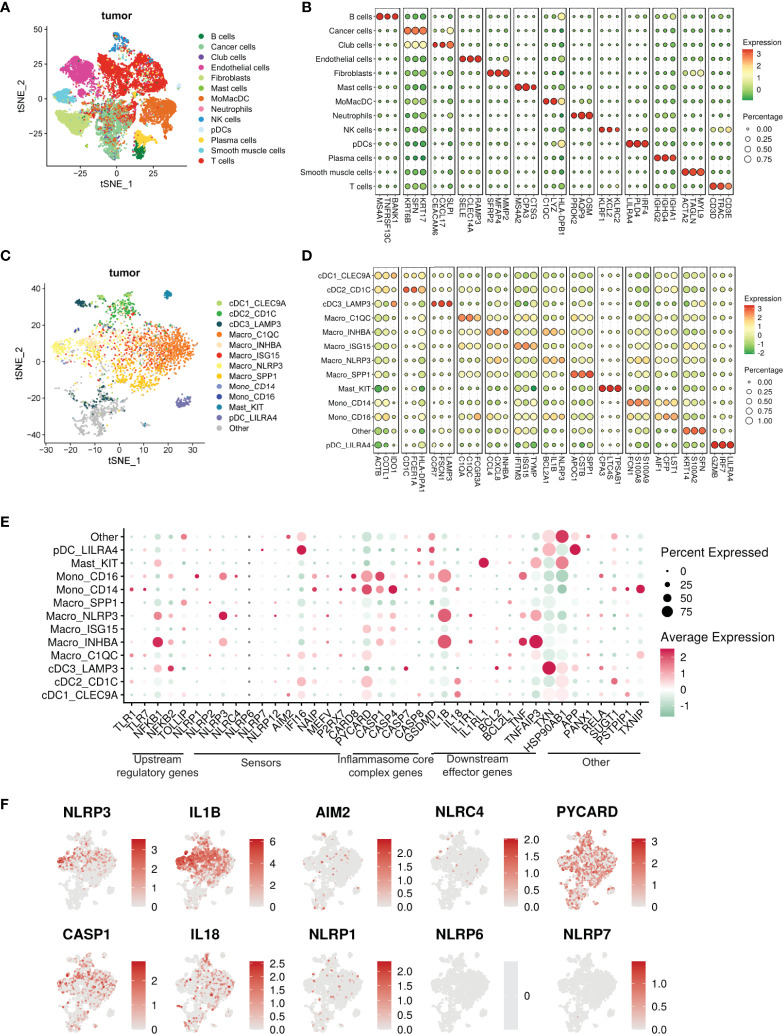
Single cell transcriptomic atlas reveals a heterogenous tumor infiltrating myeloid cell landscape with differential inflammasome signatures in each subtype. **(A)** tSNE plot showing the transcriptome landscape of 19,193 cells in the TME from patients with HNSCC (n=6). Colors indicate cell types. **(B)** Bubble heat map showing relative expression levels of typical signature genes for all cell types shown in panel (A). **(C)** tSNE showing the subtypes of 3072 myeloid cells (subset of the entire cell population) in the tumor excluding neutrophils, divided into 13 different subclusters. The “other” subset could not be characterized.”tSNE showing the phenotypic heterogeneity of 3072 myeloid cells in the tumor excluding neutrophils, divided into 13 different subclusters. The “other” subset could not be characterized. **(D)** Bubble heat map showing expression levels of selected signature genes for the tumor infiltrating myeloid cell populations shown in panel (C)**(E)** Bubble heat map showing relative expression of inflammasome pathway genes in the tumor infiltrating myeloid cell subclusters. **(F)** Feature plots showing gene expression of selective inflammasome genes in the identified tumor infiltrating myeloid cell populations. Red and gray represent high and low expression respectively.

To address the heterogeneity of tumor-infiltrating myeloid cells, we performed single-cell RNA sequencing of HNSCC tumors with matched PBMC. Unsupervised Seurat and cell-type annotation clustering analyses revealed 13 different cell subsets within the TME (see Methods and **Supplementary Table S3**). We identified 8 broadly defined tumor-infiltrating CD45^+^ immune cell subsets (**Figures 4A, B**) including *LILRA4^+^* plasmacytoid dendritic cells (pDC), *C1QC^+^* and LYZ*^+^* myeloid cells encompassing monocytes, macrophages and conventional dendritic cells (MoMacDC; mononuclear phagocytes), *CD3^+^* T cells, *KLRC1^+^* NK cells, *MS4A1^+^* (CD20) B cells, *IGHA1^+^* plasma cells, *KIT^+^* mast cells, and *OSM^+^* neutrophils, broadly consistent with recent sc-RNA seq studies from multiple cancer types (41, 42, 60–68).

To further understand the components of the large myeloid cell population, we extracted subsets of myeloid cells from the entire cell population, including MoMacDC supercluster, pDCs, and Mast cells (Neutrophils were not included due the small cell number n = 12), and annotated them with a well-defined, pan-cancer level myeloid scRNAseq dataset as reference (42). We used Cell-ID (43), a multiple correspondence analysis (MCA) based technique, to annotate the myeloid cells in our dataset. Doing so, we identified 13 different myeloid subsets in the TME and 6 in the blood (**Figures 4C, D** and **Supplementary Figures S2C, D**, **Supplementary Table S4**). Out of the 13 different myeloid populations within the tumor, we characterized 5 major cell lineages- monocytes, macrophages, conventional DC (cDC), pDC, and mast cells. Our analysis revealed 5 tumor-infiltrating macrophage subsets - Macro_C1QC (*FCGR3A/C1QC/C1QA*), Macro_INHBA (*INHBA/CXCL8/CCL4*), Macro_ISG15 (*TYMP/ISG15/IFITM3*), Macro_NLRP3 (*NLRP3/IL1B/BCL2A1*) and Macro_SPP1 (*SPP1/APOC1/CSTB*) - and 3 monocyte subsets (Mono_CD14, Mono_CD16, and Mono_CD14CD16) along with 4 tumor-infiltrating DC subsets (cDC1_CLEC9A, cDC2_CD1c, cDC3_LAMP3 and pDC_LILRA4) and mast cells (**Figures 4C, D**). The three monocyte subsets were characterized by the expression of either *CD14* or *CD16*. Classical CD14^+^ monocytes expressed high levels of the myeloid-derived suppressor cell (MDSC)-associated markers *S100A8/9* and *FCN1*, whereas the non-classical CD16^+^ subset was enriched for *LST1*, *CFP*, and *AIF1*. We also identified an intermediate CD14^+^CD16^+^ monocyte subset in low abundance in the tumor. Matched peripheral blood myeloid cells were mainly enriched in classical CD14^+^ monocytes (*LYZ, S100A9, S100A8*), non-classical CD16^+^ monocytes (*LST1, C1QC, IFITM2*), cDC2_CD1c (*CFP, HLA-DPA1, FCER1A*), and pDC_LILRA4 (IRF7, GZMB, LILRA4) (**Supplementary Figures S2C, D**).

Within the tumor, only 3 mononuclear phagocytes - *CD16^+^* monocytes, *NLRP3^+^* macrophages, and *INHBA^+^* macrophages (**Figures 4E, F**, **Supplementary Figure S2E**) – had elevated levels of *NLRP3* and *IL1B* expression. Further, we found a much stronger enrichment of *NLRP3* expression over other inflammasome pathway sensors in the tumor myeloid cells (**Figure 4F**). The *NLRP3^+^* subset also expressed high levels of genes with known tumor supportive roles like *IL10, VEGFA, TIMP1, CD300E, FPR1, SERPINA1, CCL20*, and *VCAN* compared to other mononuclear phagocytes (**Supplementary Table S4**). Similarly, the *INHBA^+^* macrophage subset co-expressed pro-tumorigenic molecules such as *IL6, TGFβ, TIMP1, CCL20, CXCL1*, and *IL10*, highlighting the highly plastic and complex nature of tumor-infiltrating myeloid cells *in vivo*, consistent with recent similar studies in other solid tumors (41, 42, 61–70). From this clustering, single sample Gene Set Enrichment Analysis (ssGSEA) (71) primarily revealed enriched IL-1 and inflammasome related pathways in *NLRP3^+^* and *INHBA^+^* macrophage populations in the tumor (**Supplementary Table S5**). ssGSEA analysis also associated both these macrophage subsets with alterations in several pathways related to carcinoma progression, T cell exhaustion, cell migration, sprouting angiogenesis, and wound healing, in agreement with tumor supportive roles reported for *NLRP3* and *INHBA* (72–75).

### Caspase-1 deletion negatively regulates myeloid cell landscape in TME

**Figure 5:**
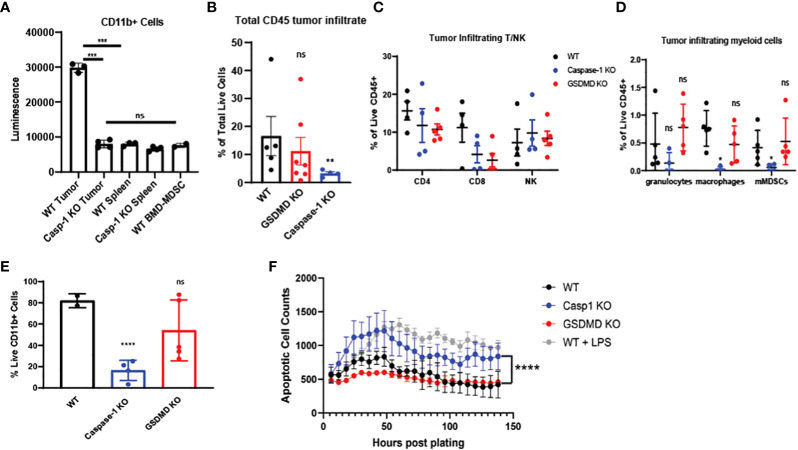
Caspase-1 deletion affects intratumoral immune landscape and myeloid cell survival in vivo **(A)** Caspase-1 activity is prominent in CD11b+ cells enriched from B16-mOVA tumors 2 weeks post tumor inoculation (n=3). CD11b+ cells were immediately utilized for caspase assay after sorting. Unstimulated mouse bone marrow derived myeloid suppressor cells were utilized as a negative control. **(B)** Caspase-1 deletion reduces global CD45+ cells in the TME compared to WT as noted by flow cytometry (n=5 mice/group, GSDMD= gasdermin D KO). B16-mOVA tumors were harvested from WT or caspase-1/GSDMD global KO mice 2 weeks post tumor inoculation. Data is first gated on the CD11b+ population then shown as the total live CD45+ cells in the total tumor. **(C)** Flow cytometry identification of tumor infiltrating T and NK cells. B16 mOVA tumors were harvested from WT or caspase-1/GSDMD null mice 2 weeks post tumor inoculation (n=5 mice/group). **(D)** Caspase-1 reduces the percentage of macrophages and monocytic MDSCs in the tumor compared to WT as noted by flow cytometry of B16-mOVA tumors (n=5 mice/group). Myeloid cell subtypes were identified according to the gating strategy outlined in **Supplemental Figure 3**. **(E)** Caspase-1 reduces the percentage of total live CD11b+ cells in the TME as noted by flow cytometry of B16-mOVA tumors. Data shown as a percentage of the total live CD11b+ cells in the tumor. (n=5 mice/group, GSDMD=gasdermin D KO). **(F)** Caspase-1 KO bone marrow derived MDSCs display higher rates of apoptosis over a 7-day time course analyzed with the IncuCyte using Annexin V as a marker of apoptotic cells and caspase 3/7 as a marker of cell death. LPS treated WT myeloid cells were used as a positive control for both markers. GSDMD KO myeloid cells were a negative control for inflammasome-mediated pyroptotic cell death. (n=3). Statistics represent one-way ANOVA with multiple comparisons **(B–E)**, two-way ANOVA **(F)**, and paired t test **(A)**. Error bars represent SD **(A–E)** and SEM **(F)**. (*p < 0.05, **p < 0.01, ***p < 0.001, ****p < 0.0001; ns, not significant).

Based on our bulk and single-cell RNA-seq analyses, we hypothesized that inflammasome signaling within the tumor infiltrating myeloid cells regulates these cells towards a tumor supportive phenotype. First, we confirmed that caspase-1 activity was significantly increased in CD11b^+^ infiltrating cells in a preclinical model (**Figure 5A**), and we directed mechanistic attention toward the tumor-intrinsic factors that regulate myeloid inflammasome signaling that was associated with tumor growth. When we examined the tumor microenvironment in caspase-1 null mice, we found that tumor infiltrating CD45^+^ cells were significantly reduced in tumors grown in these mice compared to WT mice. This was due to a significant reduction in CD11b^+^ cells, with alterations in granulocytes (MHCII^-^Ly6G^+^), macrophages (MHCII^+^CD68^+^), and monocytic MDSCs (MHCII^-^Ly6C^+^Ly6G^-^) in both B16 and MOC2 tumors (**Figures 5B–E**, **Supplementary Figures S3A, B**). While we observed a slight decrease in the tumor-infiltrating CD8^+^ and CD4^+^ T cell populations, this reduction was not significant. No alterations were found in tumor-infiltrating NK cells (**Figure 5C**, **Supplementary Figures S3A–C**). This was consistent with our previous findings that the protective anti-tumor effects of myeloid caspase-1 deficiency were not T cell dependent (4). Interestingly, tumors grown in gasdermin D null mice, which should lack the ability to undergo pyroptosis, did not phenocopy the TME profile of tumors from caspase-1 null mice (**Figures 5B–E**).

Since caspase-1 had a paradoxical effect on the CD45^+^ mononuclear phagocytes in the TME, we directly tested myeloid apoptosis in bone marrow derived myeloid suppressor cells from caspase-1 null mice. As expected, caspase-1 null cells had lower apoptosis rates, as identified by annexin V positivity and absence of caspase-3/7 staining, compared to LPS treated myeloid suppressor cells from wildtype mice, but they showed consistently higher rates of apoptosis than both untreated wildtype and GSDMD null myeloid suppressor cells (**Figure 5F**), suggesting that LPS induced apoptosis was distinct from GSDMD-dependent pyroptosis. We confirmed that these tumor-derived caspase-1 null myeloid suppressor cells could still inhibit T cell proliferation *in vitro* indicating that this pathway also does not affect their myeloid-mediated T cell suppression (**Supplementary Figures S3D–G**). Taken together, this indicates that canonical inflammasome signaling selectively regulates survival of the myeloid cell populations within in the TME.

### NLRP3/Caspase-1/IL-1β signaling axis promotes tumor growth *in vivo*

Since our single-cell analysis of human TME phagocytes showed a strong association of NLRP3 over other inflammasome sensors (**Figures 4E, F**), we performed *in vivo* tumor growth studies in NLRP3 null mice. We found that tumor growth was blunted in NLRP3 null mice, which phenocopied the previously demonstrated protective effect found in caspase-1 null mice but was unaltered in AIM2 null mice (**Figures 1A, B**). GSDMD is activated by caspase-1 to execute pyroptosis, and others suggested that GSDMD is associated with tumor growth (76). However, we did not find a tumor-supportive role for GSDMD in our models (**Figures 1A, B**), which was consistent with the immunophenotyping data from **Figures 5B–E**.

As described by others (8, 77, 78), we confirmed that the downstream mediator responsible for tumorigenesis was IL-1β as its depletion resulted in significant reduction of tumor growth (**Figures 1C, D**) which phenocopied both the caspase-1 and NLRP3 null mice. Conversely, when we treated wildtype mice with recombinant IL-1β, we saw a modest increase in tumor volume (**Figure 1E**). We found that myeloid intrinsic NLRP3-mediated activation of caspase-1 and subsequent IL-1β release is permissive towards tumor growth, and that caspase-1 regulates the survival but not trafficking of the myeloid cells to the TME.

### Efferocytosis induces *Il1b* signature in macrophages

Next, we sought to investigate the tumor intrinsic factors that can potentially activate the NLRP3-mediated inflammasome in tumor infiltrating mononuclear phagocytes. An under characterized dynamic process in the TME is efferocytosis, which is an immunologically silent phagocytic clearance of apoptotic tumor cells (10, 13, 14, 18, 21). While there have been several preclinical reports showing an association with efferocytosis and tumor progression as well as response to checkpoint blockade (9–12, 79–81), the mechanistic link between efferocytosis of tumor apoptotic cells (AC) and inflammasome activation is currently unknown.

We reanalyzed the bulk and single-cell sequencing dataset, and revealed several genes associated with distinct stages of efferocytosis were upregulated in tumor-derived CD11b^+^ cells in comparison with their PBMC counterparts (**Figure 3A**). Tumor-associated myeloid cells exhibited higher transcriptional expression of molecules linked to the “find me” (*G2A*), “eat me” (*CD93, TIM1, SCARF1, SLC2A1, GAS6, TREM2, AXL, C1QA*), and downstream processing (*NR1H3, ATG7, PPARG, ABCA1, PPARD, DOCK180*) phases of efferocytosis. Furthermore, IPA and GO analyses revealed pathways related to phagocytosis, phagosome maturation, and autophagosome organization were also significantly altered in myeloid cells isolated from the tumor (**Figure 3C**). This observation agreed with previously published studies on the involvement of the LC3 associated phagocytosis pathway (LAP) following efferocytic uptake of AC and downstream digestion (20, 82).

**Figure 6:**
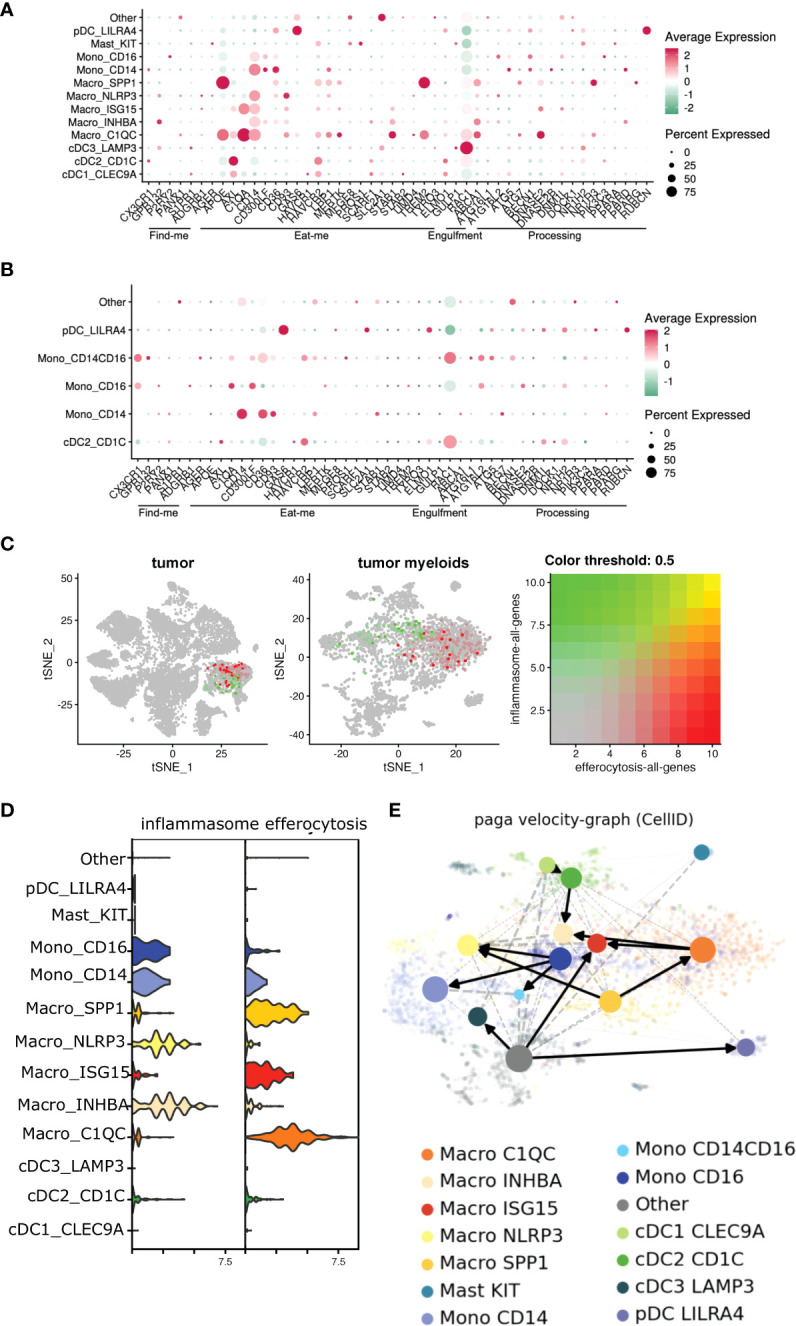
Efferocytosis gene atlas is enriched across single cell myeloid subsets in tumor and can potentially develop into inflammasome rich macrophages in the TME. **(A, B)** Single cell dataset from HNSCC patients was analyzed for genes associated with efferocytosis pathway in the myeloid subsets (n=6). Bubble heat map showing relative expression pattern of several efferocytosis genes across various myeloid subsets in the tumor **(A)** compared to matched blood **(B)**. **(C)** Feature plot shows inflammasome and efferocytosis pathway associated genes across different myeloid subsets within the tumor are not transcriptionally enriched in similar clusters. **(D)** Representative violin plot highlighting efferocytosis and inflammasome genes are enriched differentially in distinct myeloid subsets within the tumor. **(E)** Developmental trajectory of monocytes and macrophages enriched in the tumor indicate efferocytosis rich C1QC^+^ and SPP1^+^ macrophages can potentially develop into inflammasome gene rich INHBA^+^ and NLRP3^+^ subsets respectively in the TME. Cells are colored according to their cluster origin.

Single-cell dataset revealed an enrichment of multiple efferocytosis-related genes in distinct myeloid subsets within the tumor (**Figures 6A, B**), specifically, *SPP1^+^*, *ISG15^+^*, *C1QC^+^* macrophages, and *CD14^+^* monocytes in the tumors (**Figure 6D**), which were distinct from the scRNA-Seq from the peripheral blood. Our ssGSEA analyses revealed these macrophage subsets were associated with significant changes in pathways related to apoptotic cell clearance, late endosome and phagocytic vesicle, and engulfment over other non-efferocytosis gene-rich myeloid clusters (**Supplementary Table S5**). ISG15^+^ and C1QC^+^ macrophages also mobilized signaling cascades related to carcinoma and T cell exhaustion respectively. Further, ssGSEA analysis associated NLRP3^+^ and INHBA^+^ macrophage subsets with multiple pathways related to carcinoma, angiogenesis, wound healing, and T cell exhaustion; all classical hallmarks of efferocytosis (**Supplementary Table S5**) (83). Based on this, we initially hypothesized that efferocytosis in myeloid cells in the TME activates NLRP3-dependent inflammasome signaling. Interestingly, we did not observe significant overlap of these two pathways in the 8 mononuclear phagocytic populations (**Figure 6C**). However, when we performed RNA velocity analysis that imputes cell fates (53, 54, 61, 84–88), we identified a robust directional flow from the efferocytosis^high^ *C1QC^+^* macrophages towards the inflammasome^rich^ *NLRP3^+^* subset through an intermediate *ISG15^+^* macrophage state (**Figure 6E**, **Supplementary Figure S4**, **Supplementary Tables S6**). We observed that another efferocytosis-rich cluster, *SPP1^+^*, can also develop into the *NLRP3^+^* macrophage subset (**Figure 6E**). Additionally, our bioinformatic analysis showed that a fraction of the *NLRP3^+^* macrophages could arise from the *CD14^+^* and *CD16^+^* monocytes and the *INHBA^+^* macrophage subset. *NLRP3^+^* macrophages also expressed high levels of *S100A6/8/9* which in part supports their monocytic origin. Our scRNA-seq dataset further showed that NLRP3^+^ and INHBA^+^ macrophages also expressed several well-characterized immune-suppressive tolerogenic molecules (**Supplementary Tables S3**, **S4**), suggesting efferocytosis can activate a unique heterogeneous complex signature in tumor-infiltrating mononuclear phagocytes.

**Figure 7:**
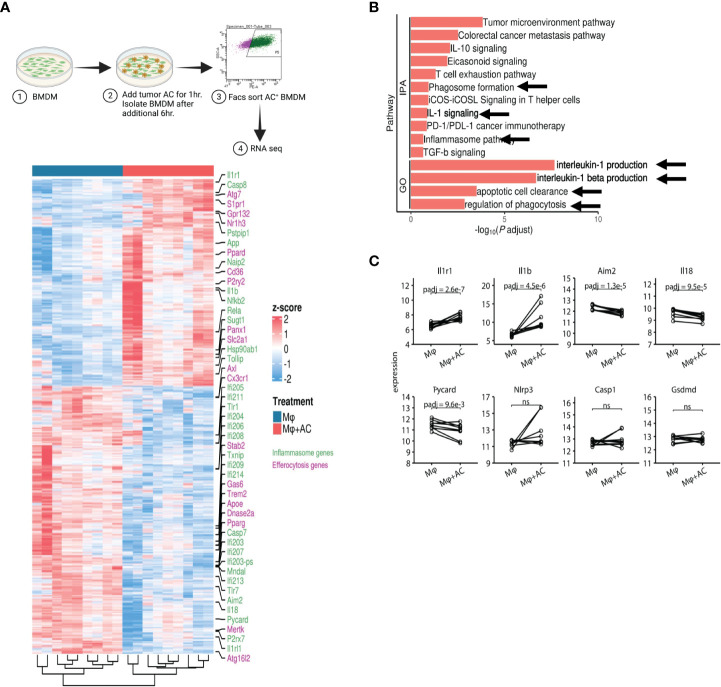
Efferocytosis in macrophages upregulates inflammasome gene expression. **(A)** Experimental workflow and heatmap for induction of efferocytosis in mouse bone marrow derived macrophages and subsequent RNA-seq (n=5). BMDM were incubated with PKH26 labeled AC for 1hr. Non-engulfed AC were washed with 1X PBS and after an additional 6hr, PKH26^+^ BMDM were sorted and processed for RNA seq. Heatmap showing differential gene expression profiles between macrophages pre and post induction of efferocytosis as measured by bulk RNA-seq. **(B)** IPA and GO analyses show increased expression of IL-1 signaling pathways post efferocytosis. **(C)** Graphical representation of selected inflammasome genes indicate enrichment of NLRP3/IL-1 pathway post efferocytosis. ns, not significant.

To directly test whether efferocytosis induces NLRP3 dependent inflammasome signaling in macrophages, we first modeled this process *in vitro*. Briefly, bone marrow derived macrophages (BMDM) were allowed to engulf tumor AC (**Supplementary Figure S5A**) stained with PKH26 dye for 1hr, washed to remove non-phagocytosed AC debris, and sorted for AC^+^ macrophages subjected to RNA sequencing (**Figure 7A**, **Supplementary Figures S5B, C**). Efferocytosis of tumor AC downregulated several well-known inflammatory genes like *nos2, il18, ccl5, cd40, cxcl10, cxcl11, cgas, ifnlr1* and *Il12rb1* and increased the expression of multiple immune suppressive and tumor-promoting genes like *tgfbi, arg2, mmp19, cd276, cxcl1, cxcl14, cxcl3, cxcl2* and *vegfa* (**Figure 7A**, **Supplementary Tables S7**) (61). This suggested that efferocytosis of apoptotic tumor cells rendered the macrophages less inflammatory and more immune suppressive in nature. Post efferocytosis, we observed a significant increase in the transcriptional activation of *Il1b* and *Il1r1* as well as a modest change in the mRNA levels of *Nlrp3* (**Figures 7A, C**), consistent with our bulk RNA-seq analysis from sorted human tumor-derived CD11b^+^ cells (**Figures 3A, B**). Several genes associated with the “find me” (*S1pr1, Gpr132, P2ry2, Panx1*), “eat me” (*Cd36, Slc2a1*) and downstream processing phases of efferocytosis (*Atg7, Nr1h3, Ppard*) were also upregulated in the efferocytic BMDM (**Figure 7A**). IPA and GO pathway analyses revealed IL-1 signaling, apoptotic cell clearance, and phagosome formation among the most significantly altered in efferocytic macrophages (**Figure 7B**). Our analyses are in agreement with existing reports highlighting the association of autophagic machinery with efferocytosis and digestion of AC as well as IL-1β secretion (20, 56–59, 82). Additionally, IPA and GO analyses showed efferocytic macrophages enriched and upregulated several tumor supportive pathways including IL-10 signaling, T cell exhaustion pathway, and PD-1/PD-L1 signaling pathways which were also enriched in our human tumor derived myeloid cell transcriptomic profiles (**Figures 3C**, **6B**) (89, 90). Previous studies on efferocytosis also show the importance of IL-10 and TGF-β in efferocytosis mediated macrophage remodeling (91, 92). Interestingly, TGF-β,PD-L1 and IL-1β can also be supportive in their functions and regulation (93–95). Targeting PD-L1 can also adversely increase NLRP3 activity and hence IL-1β secretion and drive therapy resistance in tumors (96). It is tempting to speculate that a similar crosstalk operates in efferocytic macrophages in the TME and warrants further investigation.

Taken together, our results suggest that efferocytosis of apoptotic tumor cells enhance the suppressive phenotype in macrophages characteristic of tumor associated macrophages (9–11).

### Efferocytosis of tumor AC activates NLRP3-dependent inflammasome activation and IL-1β secretion in macrophages *in vivo*

To study the efferocytosis-driven NLRP3-dependent inflammasome activity *in vivo*, we employed a commercially available ASC-citrine transgenic mouse model which allowed us to observe “speck” formation when the adaptor protein ASC oligomerizes to form a large aggregate (inflammasome activation) (97). We first incubated BMDM from the ASC-citrine/LysmCre compound mice with Annexin V^+^PI^-^ early apoptotic MOC2 cells (**Supplementary Figure S5D**, **Figure 2A**) and observed inflammasome “speck” formation in BMDM at 8 and 12 hours post efferocytosis (**Figures 2B, C**). This was associated with significant levels of IL-1β production in response to efferocytosis (**Figure 2D**) with low levels of IL-18 secretion. IL-1β has been shown to have a strong tumor-promoting role while conversely, IL-18 has anti-tumorigenic effects (8, 78, 98, 99). Here the ratio of IL-1β/IL-18 shows that IL-1β was secreted at a higher concentration than IL-18 from efferocytic macrophages indicating skewing toward a more pro-tumorigenic phenotype. Further, IL-1β production is completely diminished in the caspase-1 and GSDMD KO mice (**Figure 2D**).

When we treated BMDM with MCC950, a known small molecule inhibitor for NLRP3, we did not observe any ASC “speck” formation or IL-1β secretion in BMDM following efferocytosis (**Figures 2B–D**). Notably, MCC950 did not affect the uptake efficiency of tumor AC in macrophages (**Supplementary Figure S6A**). Importantly, we did not find inflammasome activation with recombinant IL-4 or conditioned media from live and tumor AC (**Supplementary Figure S6C**), showing that the effect is not mediated by either immunosuppressive cytokines or soluble factors released from apoptotic cells alone.

Treatment of BMDM with Annexin V beads alone failed to induce inflammasome “specks” (**Supplemental Figure S6E**) suggesting activation of the inflammasome pathway requires the presence of tumor AC. We also did not observe “speck” formation following efferocytosis in cytochalasin D treated BMDM, which prevented the engulfment of tumor AC by the macrophages, further indicating that engulfment of tumor AC is required for inflammasome activation (**Supplementary Figure S6B**, **Figures 2C, E**). In alignment with this, we did not detect any IL-1β or IL-18 secretion in Cytochalasin D treated macrophages following efferocytosis (**Figure 2D**). To rule out the possibility that Cytochalasin D alone affected “speck” formation, we incubated LPS+Nigericin treated BMDM with the same dose of Cytochalasin D used for our efferocytosis assays and saw no significant change in the average number of “speck” positive cells in these macrophages (**Supplementary Figure S6D**).

We next examined efferocytosis-induced NLRP3-dependent inflammasome activation *in situ* from MOC2 tumor-bearing ASC-citrine mice. We detected speck formation in F4/80^+^ tumor-infiltrating macrophages, which was significantly reduced upon treatment of tumor bearing mice with MCC950 (**Figure 2F**, **Supplementary Figure S7**). To further demonstrate that NLRP3/IL-1β signaling is a key mediator of tumor growth, we performed a rescue experiment in NLRP3 KO mice. We were able to rescue the pro-tumor effect of NLRP3 signaling with the addition of recombinant IL-1β (**Figure 2G**).

Finally, we wanted to confirm these findings in humans, so we next looked at the association of efferocytosis and inflammasome signaling using macrophages derived from peripheral blood. Human macrophages showed efferocytosis-dependent IL-1β secretion without the production of IL-18, and we did not see production of either cytokine in efferocytic human macrophages treated with MCC950 or Cytochalasin D (**Figure 2H**). These *in vitro* findings link efferocytosis of tumor AC and inflammasome-mediated IL-1β release in human macrophages to support our hypothesis.

Our findings support the critical role of efferocytosis of tumor AC to activate NLRP3-dependent inflammasome signaling in the TME to induce non-pyroptotic IL-1β secretion which subsequently promotes tumor growth (Graphical Abstract).

## Discussion

Our findings demonstrate for the first time that efferocytosis of apoptotic tumor cells activates myeloid-intrinsic NLRP3/caspase-1/IL-1β signaling in the tumor microenvironment. Others showed that apoptotic tumor cell clearance can be an immunologically quiescent process (25–31, 100, 101), and we now offer a mechanistic link that efferocytosis can direct canonical inflammasome signaling to enrich for immunosuppressive tumor-infiltrating macrophages and MDSCs. Although earlier reports showed that cells undergoing apoptosis or autophagic death can activate NLRP3 dependent inflammasome signaling in macrophages (35, 102), the relationship between efferocytosis of AC and inflammasome signaling was not known. While some GI and colon tumor models indicate that tumor-intrinsic inflammasome signaling may have anti-tumorigenic effects (103, 104), we have complemented our *in vivo* studies with a single cell transcriptomic profile of human squamous carcinoma that corroborates a distinct pro-tumorigenic role for the myeloid-intrinsic NLRP3-dependent inflammasome. Our investigations clearly demonstrate the mechanistic role of efferocytosis driving NLRP3-dependent inflammasome activation in the TME which helps generate a pro-tumorigenic TME.

Our report has several scientific and translational implications. First, our single-cell transcriptomic profile illustrates the heterogeneity and plasticity of the tumor infiltrating myeloid cells. Within the 5 macrophage subsets we identified, *NLRP3* and *IL1B* inflammasome gene expression were predominant in 2 subsets, NLPR3^high^ and INHBA^high^, and our RNA velocity analysis suggests that these subsets may be derived from efferocytic myeloid cells. We also identified a CD14^+^ monocyte population, which was highly enriched for MDSC-associated genes, but this population as well as the other two monocyte populations did not show an upregulation in inflammasome gene expression indicating that macrophages contribute most significantly to this mechanism. Consistent with the current knowledge that questions the dichotomic M1/M2 nomenclature of macrophages (105), we could not confirm or identify distinctly these polarized macrophage clusters in the human tumor milieu. Interestingly, we also detected high levels of *IL-1R1* expression in human HNSCC tumor infiltrating myeloid cells as well as murine macrophages post efferocytosis *in vitro*. IL-1β has been shown to act *via* its receptor and prime the inflammasome signaling pathway (signal 1). This suggests a paracrine signaling pathway where efferocytosis in one macrophage can potentially stimulate the inflammasome signaling in nearby myeloid cells, which remains to be investigated.

Our report also underscores a targetable mechanism of non-immunogenic tumor cell death distinct from the immunogenic cell death induction paradigm that has shaped current clinical trials (106–113). While we did not see a role for the DNA sensing molecule AIM2 in our studies, others have noted a myeloid-intrinsic STING-dependent mechanism to render the tumor more responsive to immune checkpoint inhibitors (114–118). Of note, while others have shown a mechanistic link between the inflammasome and STING signaling, there have been limited reports directly linking efferocytosis with the STING pathway (11, 119–121). A translational implication from these studies is that both efferocytosis-dependent blockade of inflammasome signaling and myeloid-directed targeting of STING may be required for treatment of tumors that fail T cell-directed immune checkpoint inhibitor immunotherapies. Interestingly, our RNA seq analyses using human and murine macrophages indicated mobilization of phagocytosis, autophagosome organization and phagosome maturation pathways in tumor infiltrating myeloid cells as well as post efferocytosis. Autophagy is important for both IL-1β secretion (56–59) as well as processing of the efferocytosed AC cargo in macrophages (82). It will be interesting to speculate whether drugs inhibiting autophagy or LC3-associated phagocytosis can work in synergy with IL-1β targeting molecules and warrants investigation.

Several phagocytosis targeting agents that are hypothesized to shift the immunosuppressive TME towards an activated anti-tumor phenotype are being evaluated (122–124). One example is the combination of bavituximab and pembrolizumab, which is currently in a phase 2 trial for pretreated advanced gastric or gastroesophageal junction cancer (NCT0409641). However, because efferocytosis involves many receptors, focusing on just one may be insufficient as illustrated by a compensatory increase in MerTK expression when Axl is targeted (125), highlighting the importance of simultaneously targeting multiple efferocytosis related genes or downstream effector pathways (126). Alternatively, our study suggests that instead of combining multiple efferocytosis blocking agents, targeting efferocytosis through inflammasome blockade may be more effective in reducing tumor burden. Furthermore, triple therapy targeting MerTK and PD-1 after radiotherapy-induced abscopal anti-tumor immune responses (80) indicates a possible use for targeting efferocytosis and the inflammasome post-radiotherapy.

Our previous reports demonstrated a role for caspase-1 in monocytic MDSCs as a tumor growth mediator in a T cell-independent manner to warrant IL-1β blockade in cancer therapeutics (4). Interestingly, the CANTOS trial, which enrolled over 10,000 cancer-free patients, showed that those treated with canakinumab (IL-1β blocking agent) had reduced lung cancer incidence and cancer-associated mortality (7, 8). This paved the way for multiple clinical trials (CANOPY) with canakinumab as a single agent or in combination with PD-1 blockade or chemotherapy. However, the CANOPY trial failed to meet the primary endpoints of overall survival and progression-free survival (127, 128). These clinical studies suggest that IL-1β plays an important role in early carcinogenesis in humans. Thus, therapeutic targeting of inflammasome signaling may be more effective in patients with early-stage disease or as adjuvant therapy in patients with a high risk of recurrences.

Our findings demonstrate that efferocytosis of tumor AC creates a T cell-independent tumor permissive microenvironment by activating NLRP3 dependent inflammasome signaling and IL-1β secretion. This work points to a targetable pathway for patients with myeloid cell-rich tumors that are refractory to T cell-based immunotherapy.

## Limitations of the study

Our *in vitro* functional studies do not encompass a pure population due to the highly overlapping and complex nature of myeloid cells *in vivo* and the difficulty of purifying them in the absence of reliable unique markers. Our single-cell sequencing dataset identified a previously uncharacterized and highly complex myeloid landscape in HNSCC. However, it is difficult to predict or replicate *in vitro* the effects of inflammasome-rich macrophages on the other myeloid subsets that potentially exist *in vivo* due to the inability to sort out pure populations of each of these unique cell types. The NLRP3 dependent inflammasome pathway can be activated by a wide range of stimuli. The molecular mechanism of how cancer AC interacts with and activates NLRP3 in macrophages is currently unknown and is beyond the scope of this manuscript. Lastly, since the inflammasome KO mouse models employed in this study are not lineage-specific deletions, the effect of deleting the genes in different immune cell compartments must be considered and warrants additional exploration.

## Data and code availability

### Data availability

Raw fastq files and reads count matrix of bulk RNAseq data of mouse BMDM are uploaded to GEO database under accession number: GSE200560. Reads count matrix for bulk RNAseq data of human CD11b^+^ myeloid cells, h5 files including raw count matrix, normalized expression matrix, RNA velocity splicing and unsplicing matrices and annotation information for both entire cell population and myeloid clusters can be accessed at DOI:10.5281/zenodo.6433308. For bulk and scRNA-seq raw sequencing data of HNSCC patients accessing, reasonable request can be sent to and approved by PI (Young Kim).

### Code availability

All the relevant code related to bulk and single cell RNAseq data analysis is provided *via* GitHub https://github.com/tbilab/hnscc_myeloid.

## Experimental model and subject details

### Study approval

Our study was performed in compliance with approved Vanderbilt University Medical Center Institutional Review Board protocols in accordance with the Belmont Report and US Common Rule. All subjects provided written informed consent prior to participation, and the specimens were de-identified prior to analysis. Included were HNSCC patients undergoing surgery for curative intent. Excluded were those with autoimmune diseases and chronic steroid use. Fresh tumor specimens (typically 1 cm^3^) were harvested from non-margin areas and processed in DMEM base media within 2 hours of resection. Fresh tumor samples were processed using miltenyi biotec human tumor dissociation kit and the Miltenyi GentleMACS Octo dissociator following manufacturer’s instructions. PBMC were collected from blood using Ficoll-Paque Plus, following manufacturer’s instruction. Single cell suspensions were stained and analyzed by flow cytometry the same day the samples were collected using a BD FACS Celesta.

### Mice

6-week-old female C57BL/6J wildtype were purchased from The Jackson Laboratory. *Casp1-/-*, *Aim2-/-*, and *Nlrp3*-/- male and female mice were purchased from The Jackson Laboratory and bread for colony maintenance. *Gsdmd-/-* mice were a gift from T.D. Kanneganti (St. Jude Children’s Research Hospital). R26-CAG-LSL-ASC-citrine mice were purchased from the Jackson Laboratory. These mice are Cre recombinase-dependent fluorescent reporter of inflammasome complex activation. The R26-CAG-LSL-ASC-citrine floxed allele has a CAG promoter and *loxP*-flanked STOP cassette upstream of the ASC-citrine fusion protein, all inserted into the *Gt(ROSA)26Sor* locus. These were bred with LysMcre mice obtained from the Jackson Laboratory. Following removal of the floxed-STOP cassette, the mice express a fluorescent adaptor fusion protein (ASC-citrine) in the myeloid cells that retains the function of endogenous ASC - forming assembled inflammasome complexes (specks) upon exposure to inflammasome activator(s) both *in vitro* and *in vivo.* All experiments involving animals were reviewed and approved by the Institutional Animal Care and Use Committee at Vanderbilt University Medical Center. All experiments were performed according to NIH guidelines, the Animal Welfare Act, and US Federal law.

### Cells

Cell lines used included murine melanoma cell line B16-mOVA, Cal27 and murine head and neck cell line MOC2. All cell line aliquots were obtained from ATCC and stored in liquid nitrogen when not in use. Cells were cultured with RPMI-1640 medium, with 10% FBS, 1% penicillin/streptomycin, 1% HEPES, 1% Glutamax, and 0.1% beta-mercaptoethanol. All reagents were obtained from Gibco^®^.

### Generation of human peripheral blood derived macrophages and murine BMDM

Peripheral blood was obtained from healthy volunteers. Buffy coat was isolated using Ficoll Plaque gradient. CD11b+ cells were isolated from the buffy coat using CD11b microbeads from Miltenyi Biotech following manufacturer’s instructions. Monocytes were then cultured at 37°C with 5% CO_2_ in RPMI 1640 containing 10% fetal bovine serum, 2 mmol/L glutamine, 1% HEPES, 1% Glutamax, 0.1% beta-mercaptoethanol, 100 μg/mL streptomycin, and 100 U/mL penicillin for 7days, with either 100 ng/mL GM-CSF or M-CSF. Fresh media supplemented with appropriate concentrations of GM-CSF or M-CSF was added every alternate day. To generate M1 macrophages, on day 7, cells were treated with a cocktail of 1ug/ml LPS and 20ng/ml IFN-γ for 24hrs. For M2 macrophages, on day 7, cells were treated with 20ng/ml IL-4 for 24hrs.

For generation of mouse macrophages, bone marrow from femur and tibia of mice was flushed with ice-cold PBS using a 25-gauge 30cc needle. Red blood cells (RBC) were removed using erythrocyte lysis buffer. Cells were resuspended in complete media and differentiated using either 50ng/ml GM-CSF or 100ng/ml M-CSF for 7days. Fresh media supplemented with appropriate concentration of GM-CSF or M-CSF was added every alternate day. Where mentioned, to generate M2 macrophages, on day 7, cells were treated with 20ng/ml IL-4 for 24hrs. For M1 macrophages, on day 7, cells were treated with 1ug/ml LPS and 20ng/ml IFN-γ for 24hrs.

## Data availability statement

The datasets presented in this study can be found in online repositories. The names of the repository/repositories and accession number(s) can be found in the article/**Supplementary Material**.

## Ethics statement

The studies involving human participants were reviewed and approved by Vanderbilt University Medical Center Institutional Review Board. The patients/participants provided their written informed consent to participate in this study. The animal study was reviewed and approved by Animal facility, Vanderbilt University.

## Author contributions

CL, SR, and YK developed the original hypothesis and designed experiments. CL, SR, and DG performed experiments and/or analyzed data. YW and YX analyzed bulk and single cell RNA sequencing datasets. CL, SR, MK, and YK wrote and edited the manuscript. CHS, reviewed and provided insight on the manuscript. MK and YK supervised the study. All authors contributed to the article and approved the submitted version.

## Funding

This work was supported by the Barry Baker Biorepository Fund (YK), NIH R01 CA178613 (YK), and R01 DE027749 (YK).

## Acknowledgments

We thank the Amy and Barry Baker Family Trust, VUMC Animal Care and Use Program, Dave Flaherty, and Brittany Matlock of VU FACS core for assisting with cell sorting, VU VANTAGE for bulk and 10X sc-RNA sequencing and VU CISR for flow cytometry facilities.

## Conflict of interest

YK reports grants from NCI and NIDCR during the conduct of the study, personal fees from Aduro, AstraZeneca, Sanofi, Takeda, and Mersanna outside the submitted work. In addition, YK has a patent for CAF signature pending and is currently an employee with Regeneron Pharmaceuticals.

The remaining authors declare that the research was conducted in the absence of any commercial or financial relationships that could be construed as a potential conflict of interest.

## Publisher’s note

All claims expressed in this article are solely those of the authors and do not necessarily represent those of their affiliated organizations, or those of the publisher, the editors and the reviewers. Any product that may be evaluated in this article, or claim that may be made by its manufacturer, is not guaranteed or endorsed by the publisher.
