# Supplementary material for: Efferocytosis drives myeloid NLRP3 dependent inflammasome signaling secretion of IL-1β to promote tumor growth
Source: Front Immunol. 2022 Nov 9;13:993771. doi: 10.3389/fimmu.2022.993771 (PMC9681818; doi:10.3389/fimmu.2022.993771)
Supplement: Supplementary file 1 [file DataSheet_1.pdf]

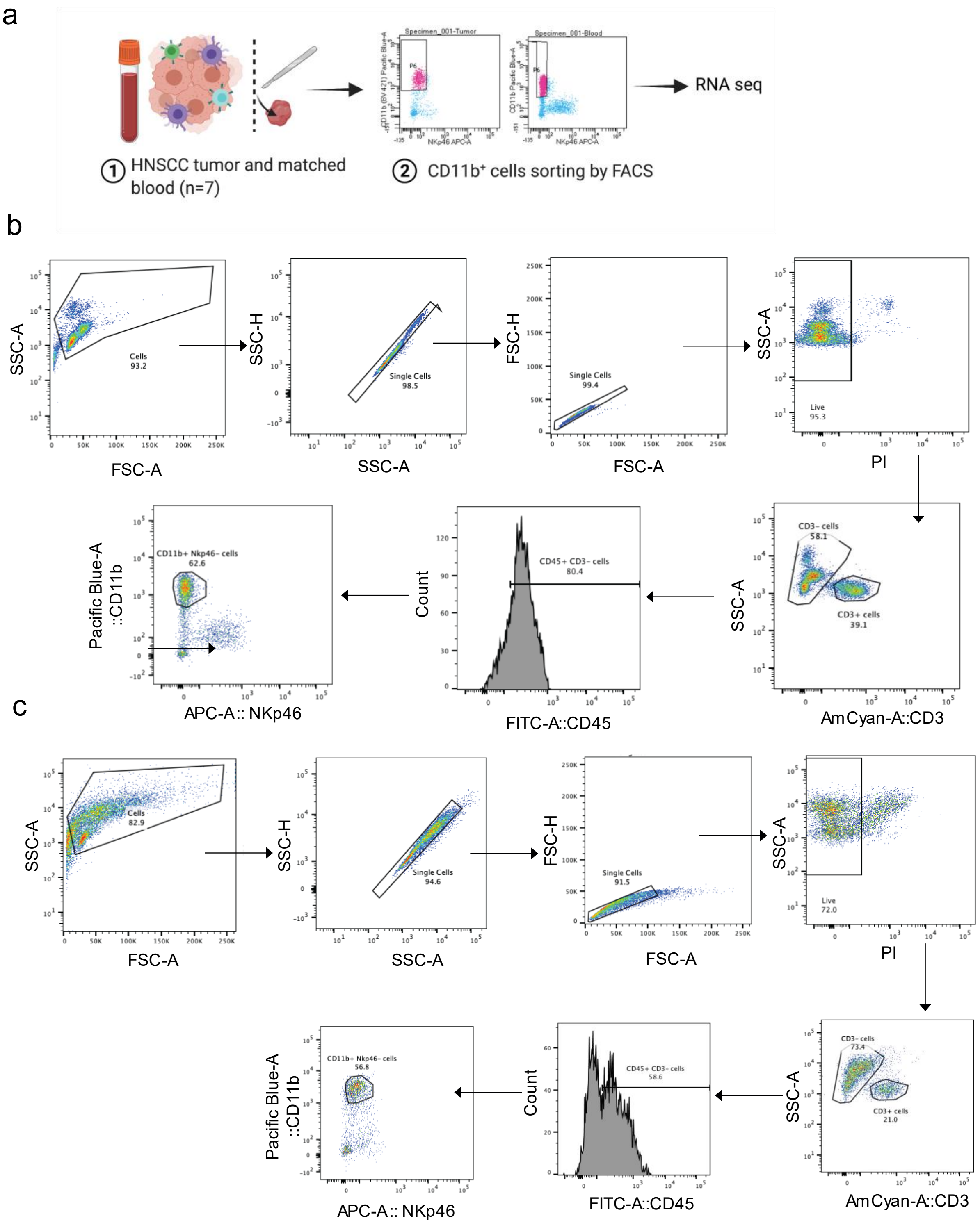
a
HNSCC tumor and matched blood (n=7)
CD11b+ cells sorting by FACS
RNA seq
b
SSC-A
FSC-A
Cells 93.2
SSC-H
SSC-A
Single Cells 98.5
FSC-H
FSC-A
Single Cells 99.4
PI
Live 95.3
SSC-A
AmCyan-A::CD3
CD3- cells 58.1
CD3+ cells 39.1
Count
FITC-A::CD45
CD45+ CD3- cells 80.4
Pacific Blue-A ::CD11b
APC-A:: NKp46
CD11b+ Nkp46- cells 62.6
c
SSC-A
FSC-A
Cells 82.9
SSC-H
SSC-A
Single Cells 94.6
FSC-H
FSC-A
Single Cells 91.5
PI
Live 72.0
SSC-A
AmCyan-A::CD3
CD3- cells 73.4
CD3+ cells 21.0
Count
FITC-A::CD45
CD45+ CD3- cells 58.6
Pacific Blue-A ::CD11b
APC-A:: NKp46
CD11b+ Nkp46- cells 56.8

Figure S1

Supplementary Figure S1, Related to Figure 1

Workflow and gating strategy for sorting CD11b$^+$ myeloid cells from HNSCC tumor and matched blood.

(**A**) Overview of the workflow for bulk transcriptomic profiling of CD11b$^+$ myeloid cells from HNSCC tumors and matched peripheral blood (n=7). (**B-C**) Single live CD45$^+$ cells were gated to exclude T cells and NK cells and pure CD11b$^+$ myeloid cells were flow sorted from (**B**) HNSCC tumor and (**C**) matched blood (n=7).

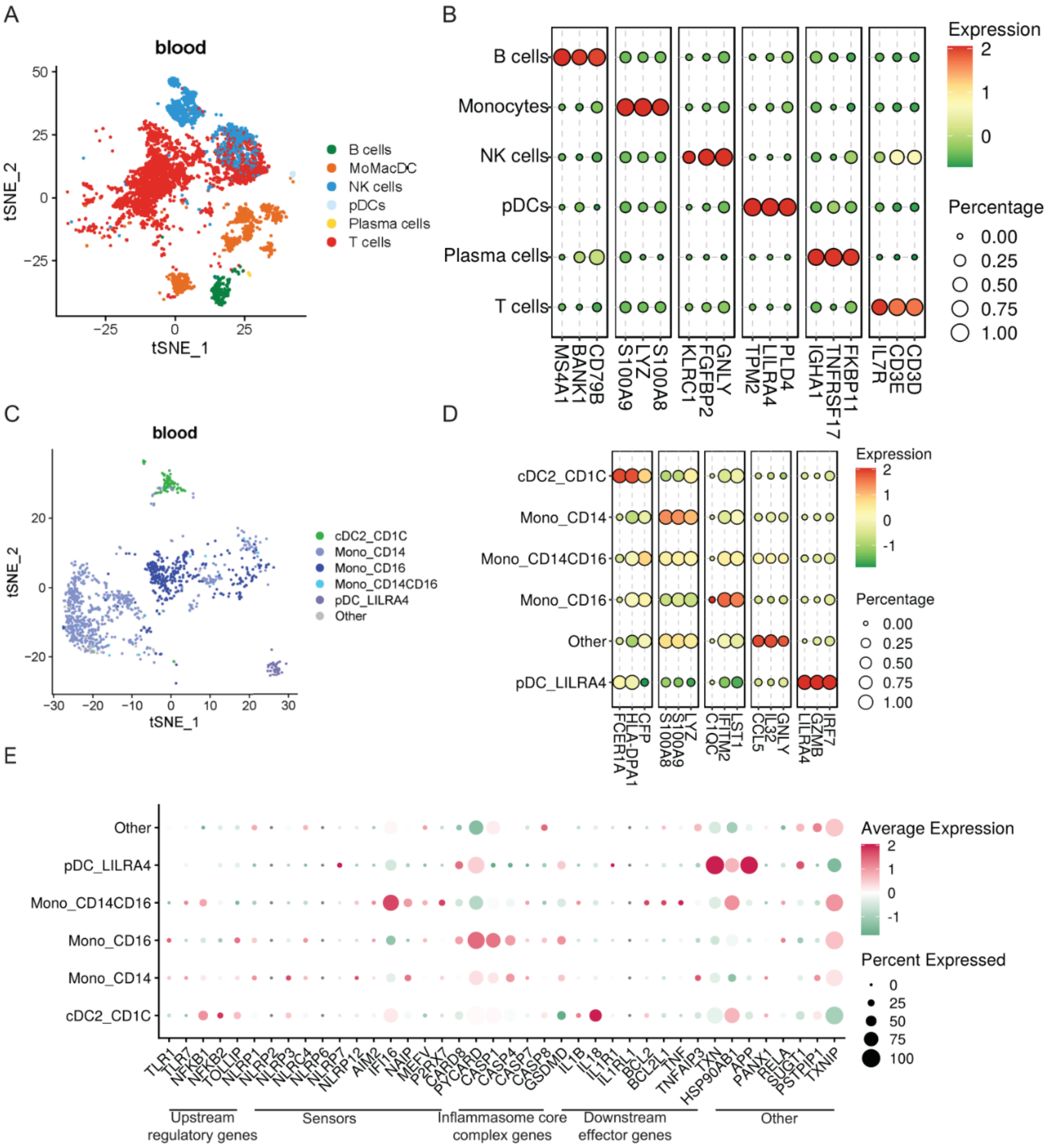
A
blood
tSNE_1
tSNE_2
B cells
MoMacDC
NK cells
pDCs
Plasma cells
T cells
B
Expression
Percentage
B cells
Monocytes
NK cells
pDCs
Plasma cells
T cells
MS4A1
BANK1
CD79B
S100A9
LYZ
S100A8
KLRC1
FGFBP2
GNLY
TPM2
LILRA4
PLD4
IGHA1
TNFRSF17
FKBP11
IL7R
CD3E
CD3D
C
blood
cDC2_CD1C
Mono_CD14
Mono_CD16
Mono_CD14CD16
pDC_LILRA4
Other
D
Expression
Percentage
FCER1A
HLA-DPA1
CFP
S100A8
S100A9
LYZ
C1QC
IFITM2
LST1
CCL5
IL32
GNLY
LILRA4
GZMB
IRF7
E
Average Expression
Percent Expressed
TLR1
TLR7
NFKB1
NFKB2
TOLLIP
NLRP1
NLRP2
NLRP3
NLRC4
NLRP6
NLRP7
NLRP12
AIM2
IFI16
NAIP
MEFV
P2RX7
CARD8
PYCARD
CASP1
CASP4
CASP7
CASP8
GSDMD
IL1B
IL18
IL1R1
IL1RL1
BCL2
BCL2L1
TNF
TNFAIP3
TXN
HSP90AB1
APP
PANX1
RELA
SUGT1
PSTPIP1
TXNIP
Upstream regulatory genes
Sensors
Inflammasome core complex genes
Downstream effector genes
Other

Figure S2

Supplementary Figure S2, Related to Figure 2

ScRNA seq reveal enrichment for NLRP3 inflammasome pathway in tumor derived CD11b$^+$ cells over matched peripheral myeloid cells

(**A**) tSNE plot showing transcriptomic atlas of 8506 different cell types identified in blood of HNSCC patients (n=6). (**B**) Top three markers for each cell type identified in blood of HNSCC patients identifying T cell subsets, NK cells, B cells, and myeloid cells. (**C**) 3489 Different myeloid subsets identified in blood of HNSCC patients (n=6) excluding neutrophils shown with (**D**) their top three markers. The "other" subset could not be characterized. (**E**) Bubble plot showing inflammasome signature across different myeloid sub clusters in the blood of HNSCC patients.

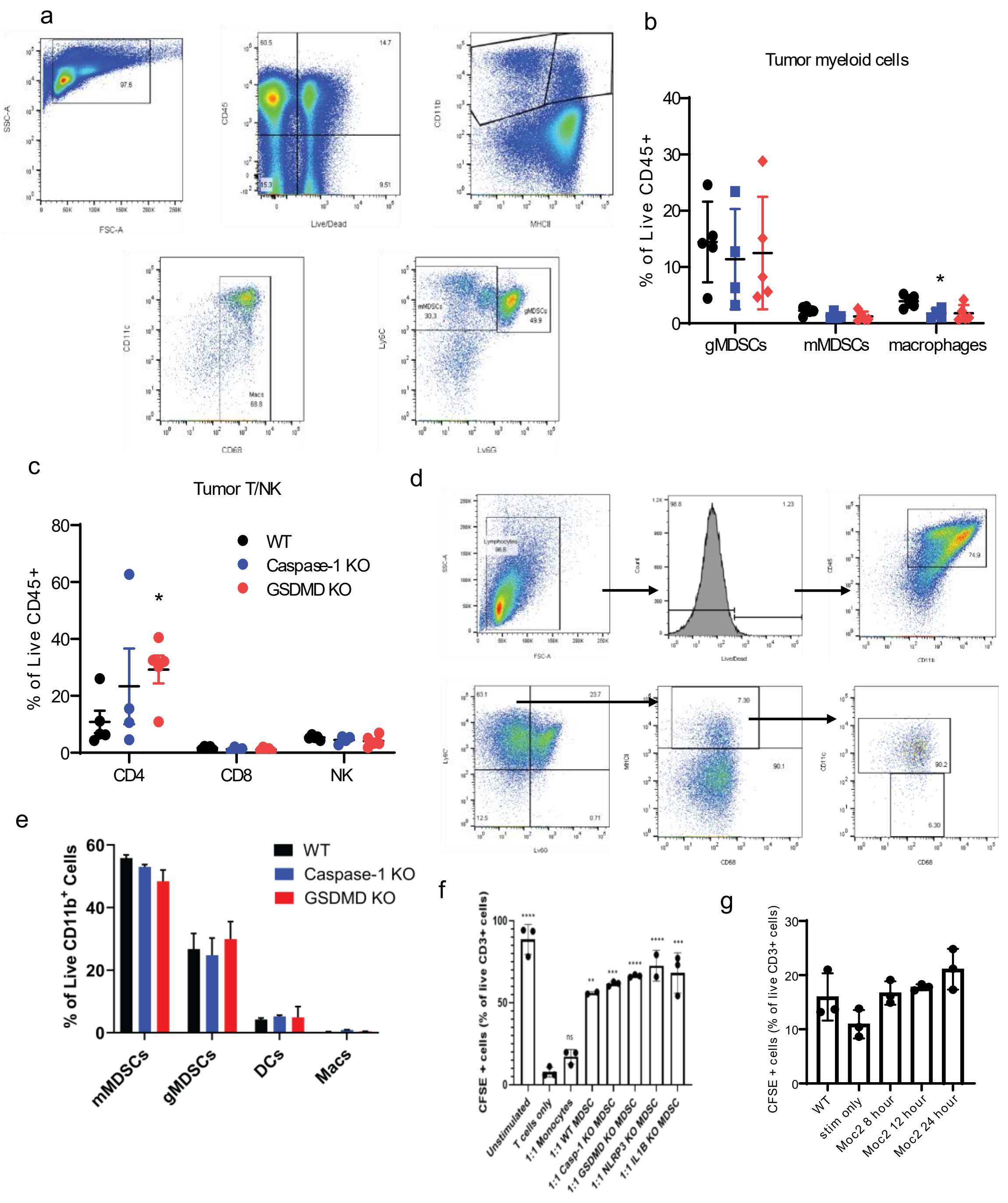

a
b
Tumor myeloid cells
% of Live CD45+
gMDSCs
mMDSCs
macrophages
c
Tumor T/NK
% of Live CD45+
WT
Caspase-1 KO
GSDMD KO
CD4
CD8
NK
d
e
% of Live CD11b+ Cells
mMDSCs
gMDSCs
DCs
Macs
f
CFSE + cells (% of live CD3+ cells)
Unstimulated
T cells only
1:1 Monocytes
1:1 WT MDSC
1:1 Casp-1 KO MDSC
1:1 GSDMD KO MDSC
1:1 NLRP3 KO MDSC
1:1 IL1B KO MDSC
g
CFSE + cells (% of live CD3+ cells)
WT
stim only
Moc2 8 hour
Moc2 12 hour
Moc2 24 hour

Figure S3

Supplementary figure S3, Related to Fig 3

Flow cytometry gating strategy for identification of tumor infiltrating myeloid cell subsets in tumor bearing mice, myeloid skewing, and T cell proliferation

(**A**) B16 tumors isolated from WT, caspase-1 KO, and gasdermin D KO mice 2 weeks post injection. Tumors were processed into single cell suspensions for analysis by flow cytometry. After gating on the Live, $CD45^+$, $CD11b^+$ population, macrophages were defined as $MHCII^+$, $CD68^+$, mMDSCs were defined as $MHCII^-$, $Ly6C^+$, $Ly6G^-$, and gMDSCs were defined as $MHCII^-$, $Ly6C^+$, $Ly6G^+$. (**B-C**) Flow cytometric analysis of wildtype MOC2 tumor infiltrating myeloid and T cells grown in wildtype C57Bl/6, caspase 1 and gasdermin D knockout mice. (n= 5 mice/group) (**D**) Flow cytometry analysis of wildtype bone marrow derived MDSCs gating strategy. Monocytic MDSCs were defined as $CD11b^+$, $Ly6C^+$, $Ly6G^-$, $MHCII^-$, granulocytic MDSCs were defined as $CD11b^+$, $Ly6G^+$, $MHCII^-$, dendritic cells were defined as $CD11b^+$, $CD11c^+$, $MHCII^+$, and macrophages were defined as $CD11b^+$, $MHCII^+$, and $CD68^+$. (**E**) Wildtype, caspase-1, gasdermin D, IL-1β, and NLRP3 knockout bone marrow skewing to mMDSCs, PMN-MDSCs, DCs, and macrophages. Bone marrow cells were isolated and cultured for 4 days in 10 ng/ml GM-CSF and bead enriched for the CD11b+ population. (**F**) Proliferation of wildtype $CD3^+$ T cells isolated from the spleens of C57Bl/6 mice co-cultured with wildtype, caspase 1, gasdermin D, IL-1β, and NLRP3 knockout bone marrow derived myeloid cells. Proliferation was analyzed as a reduction in $CFSE^+$ T cells via flow cytometry. (**G**) Proliferation of $CD3^+$ T cells was not altered when treated with supernatant from efferocytic macrophages. All data shown from independent experiments with n=3 biological replicates.

a

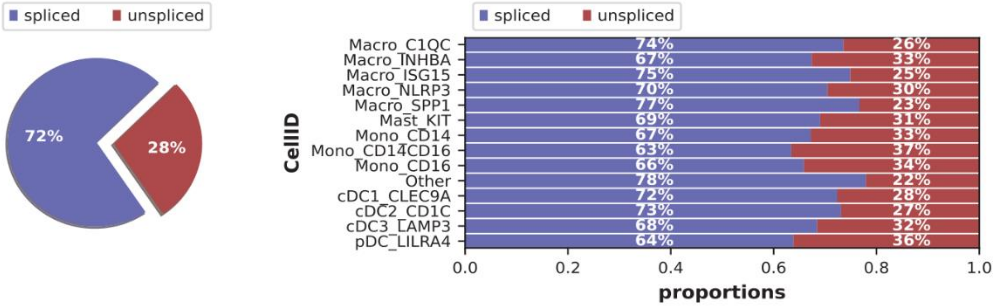
spliced
unspliced
72%
28%
Macro_C1QC 74% 26%
Macro_INHBA 67% 33%
Macro_ISG15 75% 25%
Macro_NLRP3 70% 30%
Macro_SPP1 77% 23%
Mast_KIT 69% 31%
Mono_CD14 67% 33%
Mono_CD14CD16 63% 37%
Mono_CD16 66% 34%
Other 78% 22%
cDC1_CLEC9A 72% 28%
cDC2_CD1C 73% 27%
cDC3_LAMP3 68% 32%
pDC_LILRA4 64% 36%
CellID
0.0
0.2
0.4
0.6
0.8
1.0
proportions

b

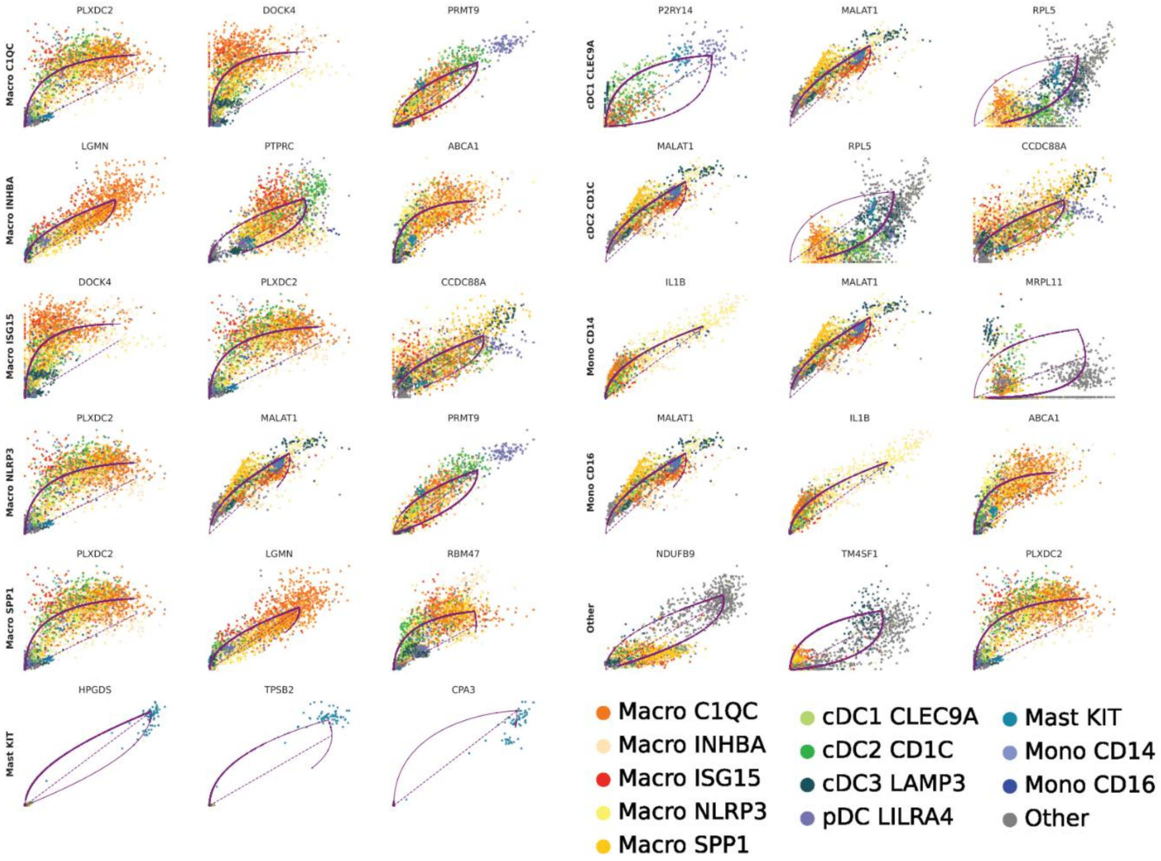
Macro C1QC
Macro INHBA
Macro ISG15
Macro NLRP3
Macro SPP1
cDC1 CLEC9A
cDC2 CD1C
cDC3 LAMP3
pDC LILRA4
Mast KIT
Mono CD14
Mono CD16
Other

Figure S4

Supplementary Figure S4, Related to Figure 5

RNA velocity analysis of tumor infiltrating myeloid subclusters

(**A**) The proportion of spliced and unsliced reads counts of entire single cell RNA-Seq dataset (left) and each subtype of myeloid cells (right). (**B**) The top 3 cluster-specific top-likelihood genes identified using the dynamic model with scVelo for each subtype of myeloid cells.

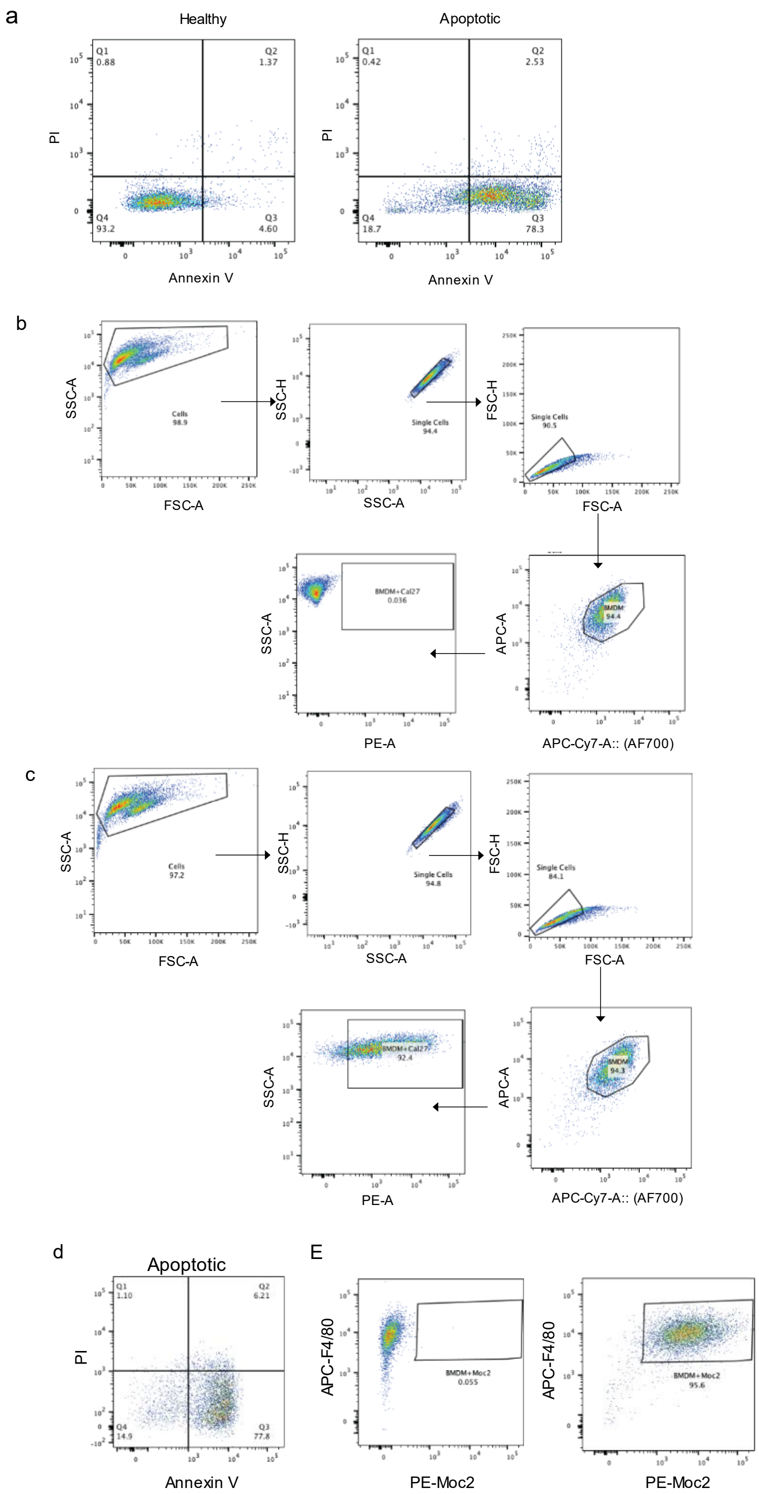

a
Healthy
Apoptotic
Q1 0.88
Q2 1.37
Q4 93.2
Q3 4.60
Q1 0.42
Q2 2.53
Q4 18.7
Q3 78.3
PI
Annexin V
b
SSC-A
FSC-A
Cells 98.9
SSC-H
SSC-A
Single Cells 94.4
FSC-H
FSC-A
Single Cells 90.5
SSC-A
PE-A
BMDM+Cal27 0.036
APC-A
APC-Cy7-A:: (AF700)
BMDM 94.4
c
Cells 97.2
Single Cells 94.8
Single Cells 84.1
BMDM+Cal27 92.4
BMDM 94.3
d
Apoptotic
Q1 1.10
Q2 6.21
Q4 14.9
Q3 77.8
E
APC-F4/80
PE-Moc2
BMDM+Moc2 0.055
BMDM+Moc2 95.6

Figure S5

Supplementary Figure S5

Induction of apoptosis in cancer cells and gating strategy for flow sorting PKH26^{+} AC^{+} BMDM, Related to Figure 6,7

(**A**) representative graph showing treatment of Cal27 with 2μM ST for 15 hours yields mostly 70-80% Annexin V^{+} PI^{-} early apoptotic cells. (**B-C**) Gating strategy for flow sorting AC^{+} BMDM post efferocytosis assay. BMDM cultured with M-CSF (100ng/ml) for 7 days were incubated with 70-80% Annexin V^{+}PI^{-} early apoptotic Cal27 cells stained with PKH26 cell labeling dye for 1 hour following which non-engulfed cells were washed away with 1X PBS. After additional 6 hours post efferocytosis, BMDM were harvested and processed for flow cytometric sorting of PKH26 AC^{+} BMDM and RNA isolation. (**D**) Representative plot showing Annexin V and PI staining in MOC2 cells treated with 75μM AZD5582 for 20 hours. (**E**) Representative uptake efficiency of apoptotic MOC2 cells by BMDM.

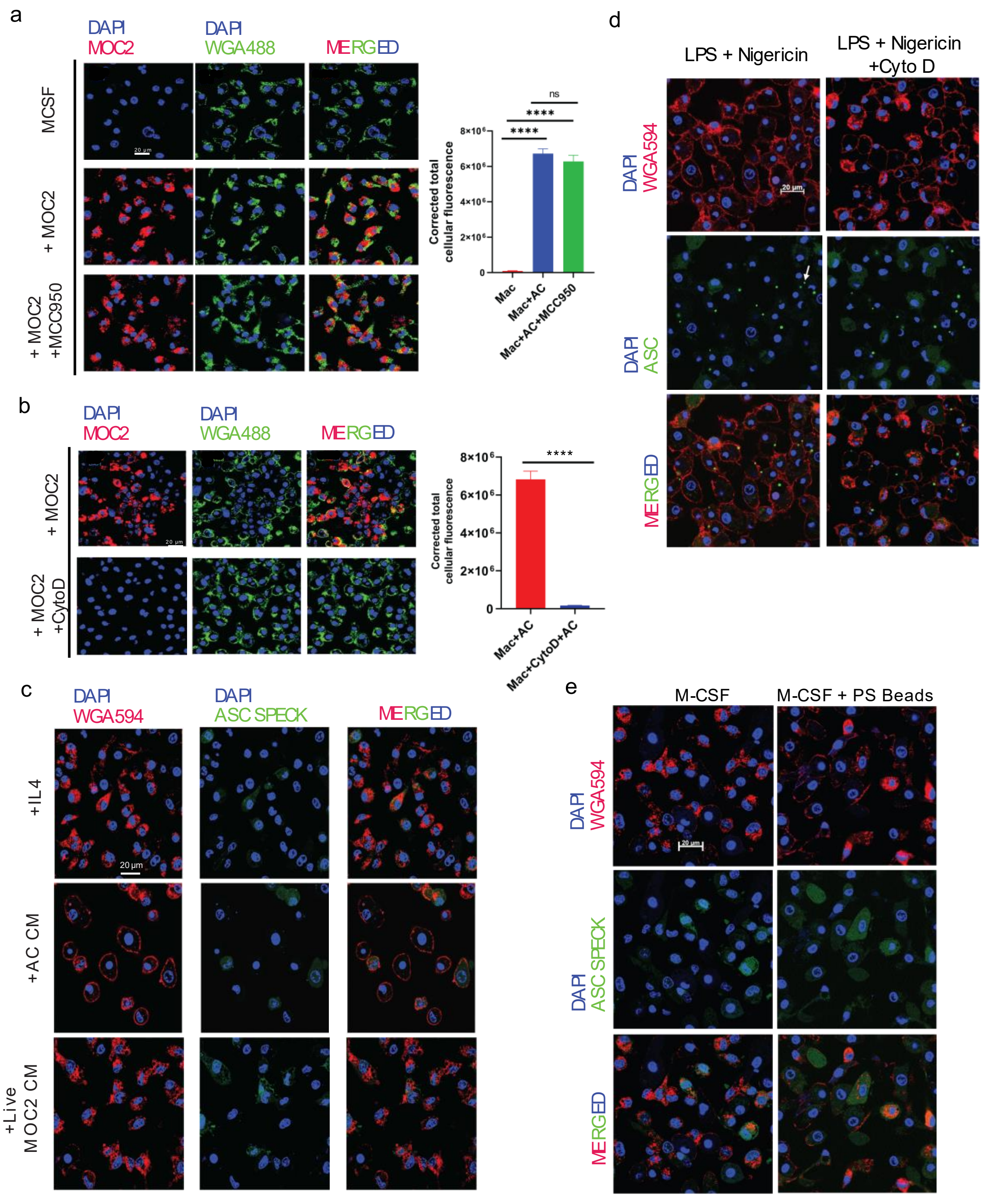
a
DAPI
MOC2
DAPI
WGA488
MERGED
MCSF
+ MOC2
+ MOC2 +MCC950
20 µm
Corrected total cellular fluorescence
8×10⁶
6×10⁶
4×10⁶
2×10⁶
0
ns
****
****
Mac
Mac+AC
Mac+AC+MCC950
b
DAPI
MOC2
DAPI
WGA488
MERGED
+ MOC2
+ MOC2 +CytoD
Corrected total cellular fluorescence
****
Mac+AC
Mac+CytoD+AC
c
DAPI
WGA594
DAPI
ASC SPECK
MERGED
+IL4
+AC CM
+Live MOC2 CM
20 µm
d
LPS + Nigericin
LPS + Nigericin +Cyto D
DAPI
WGA594
DAPI
ASC
MERGED
20 µm
e
M-CSF
M-CSF + PS Beads
DAPI
WGA594
DAPI
ASC SPECK
MERGED
20 µm

Figure S6

Supplementary Figure S6, Related to Figure 7

Effect of different conditions on uptake efficiency and inflammasome "speck" formation in BMDM

(**A**) BMDM were cultured for 7days with M-CSF and then subjected to PKH26 stained AC uptake (red) for 1 hour. Where mentioned, BMDM were treated with MCC950. Corrected total cellular fluorescence (red) was analyzed using Image J as a measure of uptake efficiency. Results are represented graphically as mean ± SEM. Data shown from n=3 biological replicates. Scale bar 20μm. (**B**) BMDM were treated with Cytochalasin D (10μM) for 1 hour to block the uptake of AC and then incubated with PKH26 labeled AC for 1 hour. Non-engulfed AC were washed away, and macrophages were stained with WGA488 (green) and DAPI (blue) used to visualize the nuclei. Corrected total cellular fluorescence (red) was analyzed using Image J as a measure of uptake efficiency. Results are represented graphically as mean ± SEM. Data shown from n=3 biological replicates. Scale bar 20μm. (**C**) BMDM grown for 7 days in the presence of M-CSF were treated with either recombinant IL-4 or conditioned media from live or apoptotic MOC2 cells and stained with WGA594 (red) cell labeling dye. DAPI (blue) was used to visualize the nuclei. Inflammasome "speck" formation (green) under these conditions were evaluated using confocal microscopy. Data shown from independent assays with n=3 biological replicates. Scale bar 20μm. (**D**) BMDM treated with LPS and IFN-γ were incubated with 10μM dose of Cytochalasin D for 1 hour (as used in efferocytosis assays). (**E**) M-CSF treated BMDM were incubated with Annexin V beads. Cells were then stained with WGA594 (red) and DAPI (blue) used for visualizing the nuclei. Inflammasome "speck" formation (green) was investigated using confocal microscopy. Top row shows BMDM (red). Middle row shows "speck" positive cells. Bottom row shows merged images

of “speck” containing BMDM. Data shown from independent experiments with n=3 biological replicates. Scale bar 20µm.

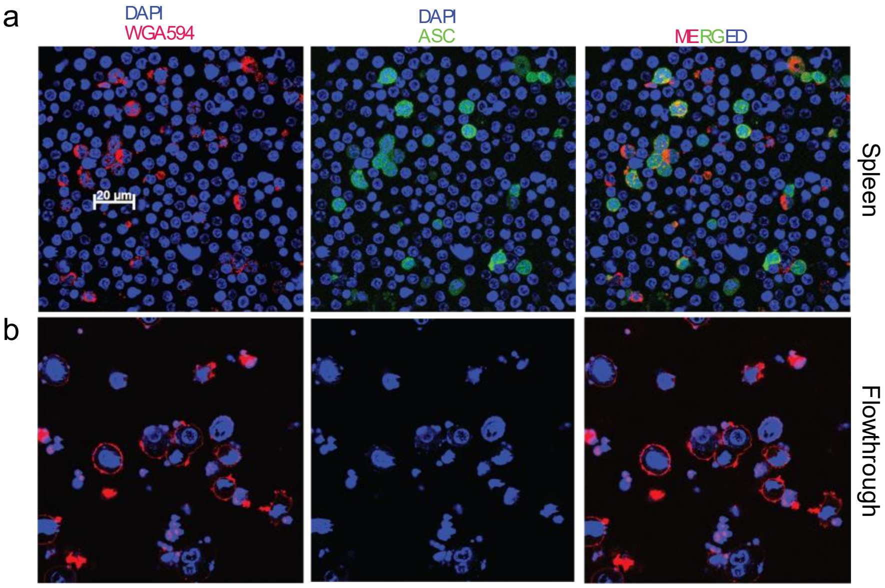

Figure S7

Supplementary Figure S7, Related to Figure 7

Splenocytes or F4/80$^-$ fraction from tumor do not exhibit "speck" positive myeloid cells

(**A**) Splenocytes harvested from the spleen of MOC2 tumor bearing ASC-Citrine/LysM Cre mice were stained with WGA594 (red) and DAPI (blue). (**B**) F4/80$^-$ fraction from MOC2 tumor grown in ASC-Citrine/LysMCre mice was harvested. Cells were stained with WGA594 and DAPI and "speck" formation was interrogated using confocal microscopy. Representative data shown from n=5 mice/group. Scale bar 20µm.

Table S1, Related to Fig 1

Significantly different expression genes between tumor and matched PBMC of HNSCC patients (FDR < 0.05).

Table S2, related to Fig 1

Collected key player genes of inflammasome and efferocytosis pathways.

Table S3, Related to Fig 2

Marker genes of major cell types identified from the single cell RNA-Seq data of HNSCC samples in blood and tumor.

Table S4, related to Fig 2

Marker genes of myeloid sub-types identified from the single cell RNA-Seq data of HNSCC samples.

Table S5, related to Fig 2

ssGSEA analysis on 10X myeloid subclusters identified in HNSCC tumor specimens (n=6).

Table S6, related to Fig 5

Cluster-specific top-likelihood genes of myeloid sub-types based on RNA velocity analysis using dynamic model.

Table S7, related to Fig 6

Significantly different expression genes between wildtype and AC treated murine macrophage (FDR < 0.05).
